# Shotgun proteomics of peach fruit reveals major metabolic pathways associated to ripening

**DOI:** 10.1186/s12864-020-07299-y

**Published:** 2021-01-06

**Authors:** Ricardo Nilo-Poyanco, Carol Moraga, Gianfranco Benedetto, Ariel Orellana, Andrea Miyasaka Almeida

**Affiliations:** 1 Escuela de Biotecnología, Facultad de Ciencias, Universidad Mayor, Camino La Pirámide, 5750 Huechuraba, Chile; 2 Université Claude Bernard Lyon 1, 69622 Villeurbanne, France; 3 Inria Grenoble Rhône-Alpes, 38334 Montbonnot, France; 4 Centro de Biotecnología Vegetal, Facultad Ciencias Biológicas, Universidad Andrés Bello, República 330, Santiago, Chile; 5 Center for Genome Regulation, Blanco Encalada, 2085 Santiago, Chile; 6 Centro de Genómica y Bioinformática, Facultad de Ciencias, Universidad Mayor, Camino La Pirámide, 5750 Huechuraba, Chile; 7 Escuela de Agronomía, Facultad de Ciencias, Universidad Mayor, Camino La Pirámide, 5750 Huechuraba, Chile

**Keywords:** Fruit ripening, Proteome, Rosaceae

## Abstract

**Background:**

Fruit ripening in *Prunus persica* melting varieties involves several physiological changes that have a direct impact on the fruit organoleptic quality and storage potential. By studying the proteomic differences between the mesocarp of mature and ripe fruit, it would be possible to highlight critical molecular processes involved in the fruit ripening.

**Results:**

To accomplish this goal, the proteome from mature and ripe fruit was assessed from the variety O’Henry through shotgun proteomics using 1D-gel (PAGE-SDS) as fractionation method followed by LC/MS-MS analysis. Data from the 131,435 spectra could be matched to 2740 proteins, using the peach genome reference v1. After data pre-treatment, 1663 proteins could be used for comparison with datasets assessed using transcriptomic approaches and for quantitative protein accumulation analysis. Close to 26% of the genes that code for the proteins assessed displayed higher expression at ripe fruit compared to other fruit developmental stages, based on published transcriptomic data. Differential accumulation analysis between mature and ripe fruit revealed that 15% of the proteins identified were modulated by the ripening process, with glycogen and isocitrate metabolism, and protein localization overrepresented in mature fruit, as well as cell wall modification in ripe fruit. Potential biomarkers for the ripening process, due to their differential accumulation and gene expression pattern, included a pectin methylesterase inhibitor, a gibbellerin 2-beta-dioxygenase, an omega-6 fatty acid desaturase, a homeobox-leucine zipper protein and an ACC oxidase. Transcription factors enriched in NAC and Myb protein domains would target preferentially the genes encoding proteins more abundant in mature and ripe fruit, respectively.

**Conclusions:**

Shotgun proteomics is an unbiased approach to get deeper into the proteome allowing to detect differences in protein abundance between samples. This technique provided a resolution so that individual gene products could be identified. Many proteins likely involved in cell wall and sugar metabolism, aroma and color, change their abundance during the transition from mature to ripe fruit.

**Supplementary Information:**

The online version contains supplementary material available at 10.1186/s12864-020-07299-y.

## Background

*Prunus persica* (L) Batsch is one of the most economically important fruit crops in the Rosaceae family, with a broad climate distribution, relatively high yield and around 1000 cultivars produced worldwide [1, 2]. *P. persica* has also become a very important plant model given its compact, small (227.3 Mb) and publicly accessible genome [3], the availability of homozygous doubled haploids, and its taxonomic proximity to other important fruit species such as apricot (*P. armeniaca*), plum (*P. salicina*), almond (*P. dulcis*) and apple (*Malus domestica*) [4].

Fruit ripening is a complex process that involves changes at multiple biochemical and physiological levels which impacts gene expression [5], proteins and metabolites abundance [2, 6]. It is the last step of the broader process of fruit development, where the fruit increases in volume and, in some species, the endocarp undergoes a hardening process, enclosing the seed in a secondary lignin-rich cell wall. During ethylene-dependent ripening, fruits transit from a photosynthetically active organ into an organ where the photosynthetic machinery is dismantled, carotenoids, sugars, organic acids and volatile compounds are accumulated, and the cell wall is loosed [7]. Overall, this conversion makes the fruit attractive for consumption as a rich source of fibers, vitamins and antioxidants, as well as its flavor, color and aroma.

Peach fruit ripening has been characterized at the molecular level in processes such as volatile and aroma production [8–10], ethylene and other hormone biosynthesis and signaling [11–13], cell wall dismantling [14], pigments biosynthesis [15, 16], and organic acids and sugars metabolism [2, 17–19]. *P. persica* transcription factors (TFs) involved in anthocyanin induction [16, 20], and ethylene biosynthesis [12, 13, 20] have also been characterized. Transcriptomic studies in *P. persica* have been used to improve the understanding of the molecular processes that underlie fruit chilling injury [21–23], and have not focused on fruit ripening.

Given the above, knowledge is still lacking about how peach fruit ripening is orchestrated at the molecular level. Proteome is a highly dynamic model for understanding the biological processes in an organ. Two-dimensional (2D) gels followed by mass spectrometry (MS) analysis have been the most frequent approach to evaluate changes in the proteome of fruits undergoing ripening. However, this approach is limited by the low numbers of proteins of interest identified, co-migration of proteins within the same spot, the absence of hydrophobic proteins, the expertise required to generate high quality 2D-gels and the extended time required to perform the images assessment and statistical analysis [24, 25]. We propose that a 1D-gel followed by LC/MS-MS analysis proteomics approach based on a robust experimental and statistical framework can provide information regarding pathways and biological processes that are crucial for peach fruit ripening. Through this technique we could expand the number of proteins identified during the transition from mature to ripen fruit. The variety selected was O’Henry, since it is a proteome and transcriptome characterized melting peach variety [26, 27], which is the parental to several new-varieties.

## Results

### Mature and ripe mesocarp peach fruit proteomes differ greatly between amongst themselves and with other fruit developmental stages

**Fig. 1:**
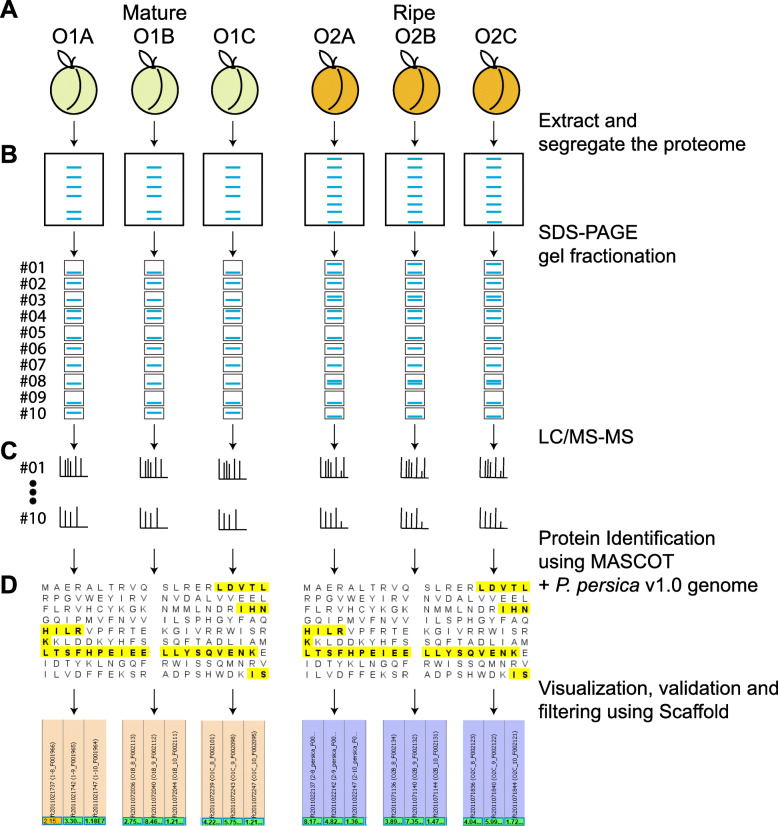
Proteomics shotgun approach used to uncover proteins involved in peach fruit mesocarp ripening process. **a** Mesocarp proteins were extracted from three biological samples (OH1A-C, OH2A-C) of mature and ripe ‘O’Henry’ peach fruit. **b** Proteins were separated using SDS-PAGE gels and later fractioned in 10 gel slices. **c** The proteins present in each slice were sequenced through LC/MS-MS. **d** The identity of the peptides present in each gel slice was assessed using Mascot and the genome sequence from *Prunus persica* v1.0. Identified proteins were further assessed using Scaffold (version 4.8.2) to identify those proteins associated with highly confident peptides and to export appropriate data for subsequent quantitative analysis (see Methods section)

The transition from mature into ripe fruit entails major changes in the fruit mesocarp firmness, titratable acidity, total soluble solids and respiration rate; and more subtle changes in ethylene biosynthesis [28]. Significant change in the respiration rate was detected in ripe fruits (83.4 mL CO_2_ kg^− 1^ h^− 1^) compared to mature fruit (15.3 mLCO_2_ kg^− 1^ h^− 1^) [28]. Ethylene production in ripe fruits (2.9 mL C_2_H_4_ kg^− 1^ h^− 1^) was the double of that measured at harvest (1.7 mL C_2_H_4_ kg^− 1^ h^− 1^), suggesting a climacteric stage. This stage is similar to the stage identified as S4II by Pan et al. [29] when ethylene autocatalytic production is increasing. After 6 days of shelf life at 20 °C, the ripe peaches showed a consistent reduction in firmness from around 60 N to 11 N. Total soluble solids were 11% at harvest and did not change after ripening [28]. To get a deeper insight into the proteins that are involved in this transition, a 1D SDS-PAGE gel followed by MS analysis was performed (Fig. 1). Data was then analyzed using MASCOT and Scaffold to focus on those proteins identified at a high confidence and to retrieve abundance data from these proteins in a format that is robust for downstream quantitative statistical analysis (Fig. 1, Supplementary Fig. 1). At first, a total of 131,435 spectra could be matched to 2740 proteins, using the peach genome reference v1. After data cleaning from proteins spuriously identified or with an inadequate number of replicates, a total of 1663 proteins were thus included in this study, representing a 6.2% of the *P. persica* primary transcripts proteome (Supplementary Table 1).

In order to check if the proteome represented by these 1663 proteins had some bias in terms of its physicochemical composition, protein properties such as length, molecular weight, charge, protein stability, and hydrophobicity profiles were compared to the *P. persica* primary transcripts proteome (26,873 proteins) and to a similar sized 977 proteins set, extracted from juicy and mealy fruit mesocarp from the Spring Lady variety [30]. In terms of data location, results indicated that for most of the parameters selected, the three populations have similar mean values (Supplementary Table 2, Supplementary Fig. 2), with the most substantial differences being related to charge and hydrophobicity, using the Guy scale [31]. In terms of data dispersion, the *P. persica* primary transcripts proteome displayed a higher dispersion for 5 of the 6 parameters assessed, when compared to the datasets from the current proteome and the proteome from Spring Lady (Supplementary Table 2, Supplementary Fig. 2).

**Fig. 2:**
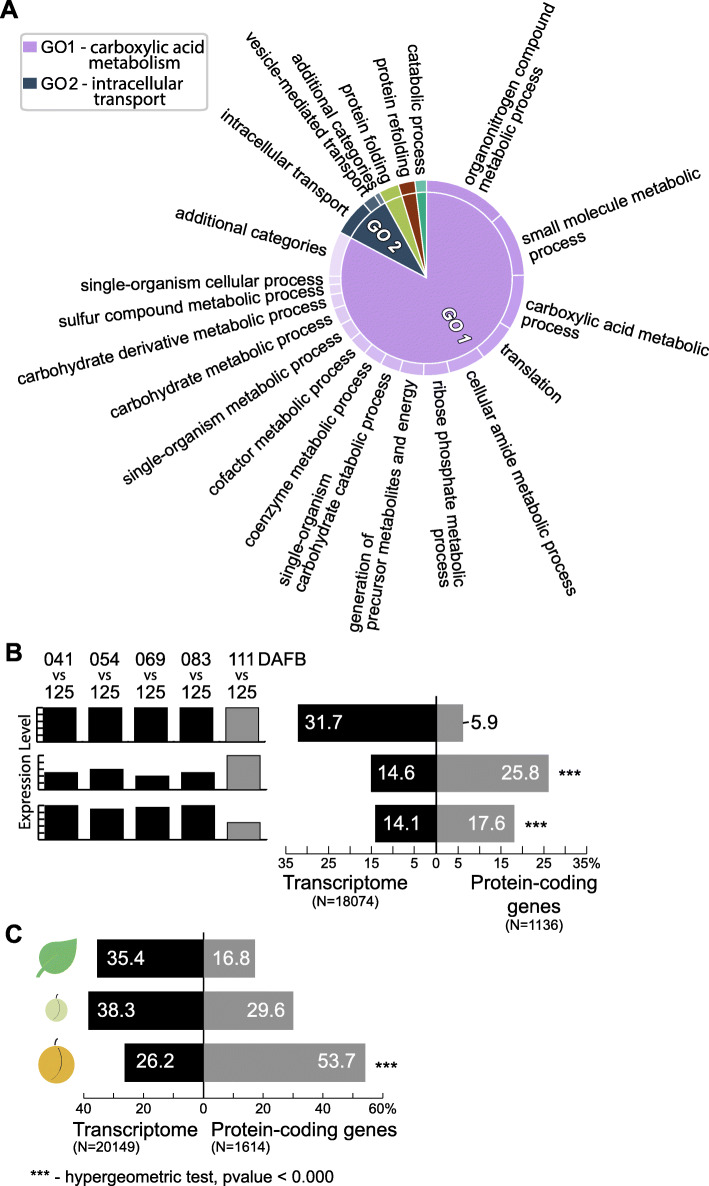
Analysis of global patterns in the proteomics dataset and comparison with related transcriptomic data. **a** Gene Ontology analysis of the 1163 proteins assessed indicated that this set was enriched in carboxylic acid metabolism, intracellular transport, and to a lesser extent in protein folding. **b** Expression levels from 18,074 genes from the “Fantasia” variety were assessed at 41, 54, 69, 83, 111 and 125 (ripe fruit) days after full bloom (DAFB). The analysis indicated that 31.7% of the genes did not display any change when comparing each developmental stage against ripe fruit, 14.6% displayed its highest level and 14.1% its lowest level at ripe fruit stage (black bars, first, second and third lane, respectively). When assessing the protein-coding genes detected in mature + ripe fruit (present study), it was estimated that 5.9% did not display any change when comparing each developmental stage against ripe fruit, 25.8% displayed its highest level and 17.6% displayed its lowest level at ripe fruit stage (grey bars, first, second and third lane, respectively). The last two analyses in ripe fruit yielded statistically significant results. **c** Expression levels from 20,149 genes of the “Babygold” variety were assessed in leaves, immature and ripe fruit. Genes that were more expressed in leaves compared to immature and ripe fruit tissues using the full transcriptome dataset accounted for 35.4% of the genes (black bars). This number dropped to 16.8% when using the proteomics dataset (grey bars). The same kind of comparison performed on the genes that were more expressed in the immature and ripe fruit yielded percentages of 38.3% vs 29.6 and 26.2% vs 53.7%, being the difference found in ripe fruit statistically significant

The functional analysis of the 1663 proteins identified in this work, performed using Gene Ontology (GO) analysis, indicated that this protein set was enriched in processes related to carboxylic acid metabolism and intracellular transport, and to a lesser extent to protein folding (Fig. 2a).

We next asked if the genes encoding the proteins present in the mesocarp of mature and ripe fruits were more expressed at this stage, or if they were expressed at the same levels in any developmental stage of the fruit development. In order to answer this question, a contrast between the transcriptional expression was performed using 18,074 *P. persica* genes (herein “full-dataset”, variety Fantasia, GEO dataset GSE71561) at 125 days after full bloom (DAFB) against its expression at 41, 54, 69, 83, 111 DAFB. Close to 32% of the “full-dataset” did not display any expression level difference when contrasting its expression at 125 DAFB, corresponding to mature-ripe fruit stage against any other stage (first lane, dark bar, Fig. 2b). This proportion was 5.9%, when considering the protein-coding genes assessed in this study (1136 genes that matched our dataset, herein “proteomics-dataset”, first lane, grey bar, Fig. 2b). When assessing the number of genes that displayed its highest expression levels at 125 DAFB (second lane, Fig. 2b), 14.6% of the “full-dataset” displayed this behavior, compared to 25.8% of the “proteomics-dataset.” Finally, when assessing the number of genes that displayed its lowest expression levels at 125 DAFB (third lane, Fig. 2b), 14.1% of the “full-dataset” displayed this behavior, compared to 17.6% of the “proteomics-dataset”. This result indicates that an important proportion of the protein-coding genes characterized in the mesocarp of the mature-ripe fruit reached their highest expression levels at the mature-ripe stage of the peach fruit developmental curve.

Similar to the previous analysis, we evaluated if the protein-coding genes expressed at the fruit mesocarp during the fruit ripening were mainly expressed at this tissue or if they had an even expression across leaves, immature and ripe fruit. Therefore, a transcriptomic dataset derived from the variety Babygold, consisting of 20,149 genes, whose expression was characterized by means of RNA-seq at leaves, 2 cm immature and ripe fruit mesocarp (Fig. 2c) [32], was assessed. Gene expression at leaves was characterized by 35.4% of the genes being more expressed at this tissue, compared to 16.8% in the current “proteomics-dataset.” Gene expression in immature fruit displayed 38.3% of the genes being more expressed at this tissue, whereas in the “proteomics-dataset” this number was of 29.6%. Finally, at ripe fruit, 26.2% of the genes displayed a higher expression in this tissue, whereas in the “proteomics-dataset” this number doubled to 53.7%. This result indicates that half of the protein-coding genes characterized in the mesocarp of the mature-ripe fruit reached their highest expression at this stage.

### Proteomic differences between mature and ripe fruits points to the mature fruit as the main stage in which sugar metabolism is modulated in the fruit mesocarp

**Fig. 3:**
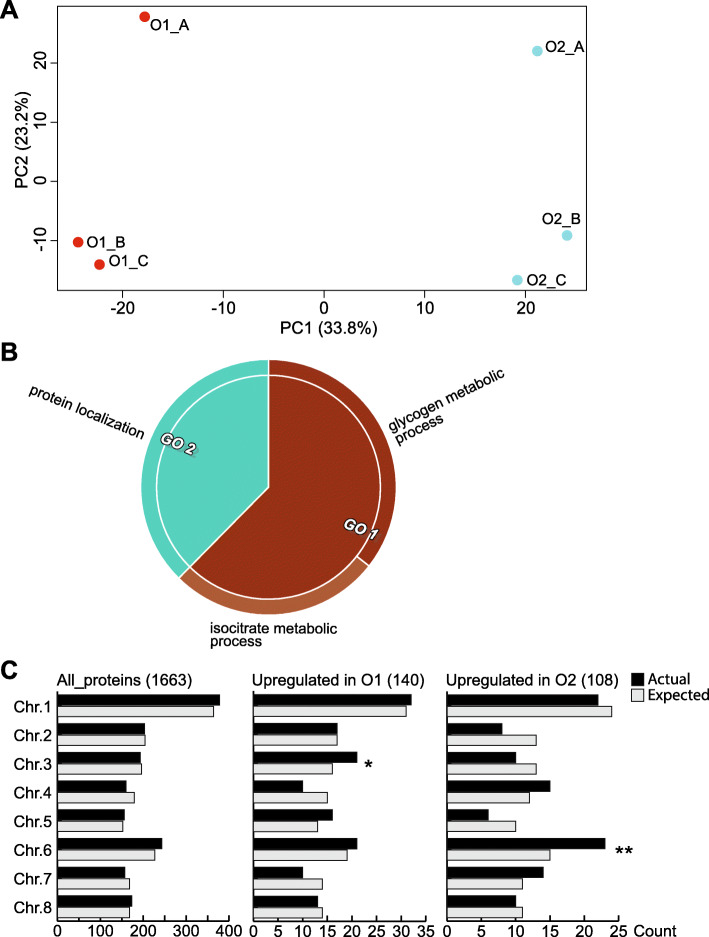
Analysis of proteins differentially accumulated in mature and ripe fruit. **a** All 1663 proteins detected in mature (O1) and ripe (O2) fruit were used to perform a Principal Component Analysis (PCA), with PC1 segregating mature from ripe fruit (**A**). From these 1663 proteins, 248 displayed a differential accumulation in O1 and O2 samples. **b** The main biological processes related to the differentially accumulated proteins in O1 fruit were associated to glycogen and isocitrate metabolism, and protein localization. **c** When assessing all the 1663 characterized proteins (“All_proteins”), no enrichment was found. Among the proteins upregulated in mature fruit, those with protein-coding genes in chromosome 3 (actual, black bars) were more than expected by chance (expected, grey bars, hypergeometric test, pvalue < 0.05). The same analysis determined that those protein-coding genes upregulated in ripe fruit were located, more than expected by chance, in chromosome 6 (pvalue < 0.01). Chr. - Chromosome

Principal component analysis (PCA) was used as a diagnostic plot and to identify the main variables that explain the proteomic differences between the samples assessed. PCA was performed using the 1663 proteins identified in all mature (O1) and ripe (O2) fruit samples assessed in this study. The principal component 1 (PC1) segregates mature from ripe fruit, explaining 33.8% of the variance associated to the samples, a high value considering that the samples used were biological replicates harvested from field grown trees (Fig. 3A). In fact, the second component could explain 23.2% of the variability and was likely associated with differences among fruits.

Quantitative changes between mature and ripe fruit proteome were assessed using a t-test after centering, normalizing and scaling the data to achieve a close to normal distribution (Methods section, Supplementary Fig. 1). Mature and ripe fruit displayed 52 and 22 proteins with an exclusive presence (referred as “Only pres.”) in each stage respectively, and 88 and 86 proteins with a significative change in abundance during the mature to ripe transition, respectively. Overall, 248 (14.9%) of the proteins identified in this study displayed a differential abundance between mature and ripe fruit (Table 1). A functional enrichment analysis of the proteins with a differential abundance was performed using Gene Ontology (GO). Proteins more abundant in mature fruit were associated to glycogen and isocitrate metabolism, and protein localization (Fig. 3B). No enrichment in any of the three GO sub-ontologies was found for proteins more abundant in ripe fruit. In terms of the chromosome distribution of the gene coding for these differentially accumulated proteins, the set differentially accumulated in mature fruit was enriched in chromosome 3; whereas the set differentially accumulated in ripe fruit was enriched in chromosome 6 (Fig. 3C). No enrichment was found after assessing all 1663 proteins identified in this study.

**Table 1:** Proteins differentially accumulated and with functional assignment

Process	Prupe_ID	Protein identification probability	Exclusive unique peptide count	DA_class^a^	Annotation	Best ***A. thaliana*** Match	Possibly Related to Volatiles Metabolism^b^
**Upregulated O1**							
***Abiotic Stress***	Prupe.4G138700	0.997	1	Up_O1	Elongation factor 2	AT1G56070	
	Prupe.6G353600	0.154	0	Up_O1	Translational activator GCN1	AT1G64790	
	Prupe.3G043000	0.998	2	Up_O1	Developmentally-regulated G-protein 2	AT1G72660	
	Prupe.5G098100	0.643	1	Up_O1	Glutathione S-transferase T1	AT5G41210	
***Actin Organization/Signaling***	Prupe.6G320500	0.995	1	Up_O1	capping protein (actin filament) muscle Z-line, alpha (CAPZA)	AT3G05520	
***Amino Acids Metabolism***	Prupe.7G039100	0.366	0	Qualit_O1	Glutamate synthase (ferredoxin) / Ferredoxin-dependent glutamate synthase	AT5G04140	
	Prupe.5G056900	1	2	Qualit_O1	Glutamate dehydrogenase 2	AT5G07440	
	Prupe.5G171400	0.998	2	Up_O1	Anthranilate phosphoribosyltransferase / Phosphoribosyl-anthranilate pyrophosphorylase	AT5G17990	
	Prupe.6G249100	0.999	1	Up_O1	Diaminopimelate epimerase, chloroplastic	AT3G53580	
	Prupe.8G013600	1	2	Up_O1	ATP phosphoribosyltransferase / Phosphoribosyl-ATP pyrophosphorylase	AT1G09795	
***Carbohydrate Metabolism/Abiotic Stress Response***	Prupe.3G289900	1	2	Qualit_O1	GALACTINOL--SUCROSE GALACTOSYLTRANSFERASE 5-RELATED	AT5G40390	
	Prupe.7G248600	1	13	Qualit_O1	GALACTINOL--SUCROSE GALACTOSYLTRANSFERASE 6-RELATED	AT5G20250	
	Prupe.6G032400	0.115	0	Up_O1	Galactinol--sucrose galactosyltransferase / Raffinose synthase	AT1G55740	
***Carbohydrates Metabolism***	Prupe.3G192600	1	7	Qualit_O1	GLUCOSE-1-PHOSPHATE ADENYLYLTRANSFERASE SMALL SUBUNIT, CHLOROPLASTIC	AT5G48300	
	Prupe.6G076300	1	3	Qualit_O1	Triose-phosphate isomerase / Triosephosphate mutase	AT3G55440	
	Prupe.1G196700	1	5	Qualit_O1	Probable fructokinase-2	AT1G06030	
	Prupe.1G354000	1	2	Qualit_O1	1,4-alpha-glucan-branching enzyme 1, chloroplastic/amyloplastic	AT5G03650	
	Prupe.1G376200	1	6	Up_O1	GLUCOSE-1-PHOSPHATE ADENYLYLTRANSFERASE LARGE SUBUNIT 2, CHLOROPLASTIC	AT1G27680	
	Prupe.1G378500	0.998	2	Up_O1	SUGAR TRANSPORTER ERD6-LIKE 4-RELATED	AT1G75220	
***Carbohydrates/Energy Metabolism***	Prupe.4G124500	0.998	2	Qualit_O1	Isocitrate dehydrogenase [NADP]	AT1G54340	
	Prupe.3G288200	0.15	0	Up_O1	ISOCITRATE DEHYDROGENASE [NADP], CHLOROPLASTIC/MITOCHONDRIAL	AT5G14590	
***Carbohydrates Metabolism/Redox Metabolism***	Prupe.2G091600	0.5	1	Up_O1	Malate dehydrogenase (NADP(+)) / NADP-linked malate dehydrogenase	AT5G58330	
***Carbohydrates Metabolism/Signaling***	Prupe.4G110600	0.995	1	Up_O1	Phosphatase IMPL1, chloroplastic	AT1G31190	
***Cell Division***	Prupe.8G209400	0.283	0	Up_O1	CLIP-associated protein	AT2G20190	
***Cell Wall Metabolism***	Prupe.5G123800	0.998	2	Qualit_O1	CELLULOSE SYNTHASE-LIKE PROTEIN G1-RELATED	AT4G23990	
	Prupe.5G118000	0.999	2	Qualit_O1	ENDOGLUCANASE 19-RELATED	AT1G64390	
***Cell Wall Metabolism/Signaling***	Prupe.7G266700	0.122	0	Qualit_O1	Probable phosphoinositide phosphatase SAC9	AT3G59770	
***Cellular Response to Light Intensity***	Prupe.3G235100	0.998	2	Up_O1	Photosystem II repair protein PSB27-H1, chloroplastic	AT1G03600	
***Cellular Response to Light Intensity/Abiotic Stress Response***	Prupe.1G264900	0.998	2	Qualit_O1	Glutathione S-transferase	AT1G10370	
***Chloroplast Photorelocation Movements***	Prupe.1G498000	1	3	Up_O1	PLASTID MOVEMENT IMPAIRED1	AT1G42550	
	Prupe.1G263000	1	2	Up_O1	protein phosphatase 2A-2	AT1G10430	
***Chloroplast protein import***	Prupe.1G170300	1	3	Up_O1	Protein TIC 22-like, chloroplastic	AT3G23710	
***Cofactor (Vit E) Metabolism***	Prupe.1G023700	0.074	0	Up_O1	2-methyl-6-phytyl-1,4-hydroquinone methyltransferase, chloroplastic	AT3G63410	
***Cytoskeleton Organization***	Prupe.7G059400	0.479	0	Up_O1	Villin-2	AT2G41740	
***Detoxification***	Prupe.4G243800	0.998	2	Qualit_O1	ADP-ribose diphosphatase / ADPR-PPase // NAD(+) diphosphatase	AT4G25434	
***Energy Metabolism***	Prupe.2G325400	1	2	Qualit_O1	Probable NADH dehydrogenase [ubiquinone] 1 alpha subcomplex subunit 12	AT3G03100	
	Prupe.1G231900	1	3	Up_O1	NADH dehydrogenase (ubiquinone) flavoprotein 1 (NDUFV1)	AT5G08530	
	Prupe.2G281900	0.998	2	Up_O1	ENOLASE	AT2G36530	
	Prupe.3G056600	1	10	Up_O1	6-PHOSPHOFRUCTOKINASE 1-RELATED	AT4G26270	
***Esters Catabolism***	Prupe.1G439300	0.998	2	Up_O1	CARBOXYLESTERASE 2-RELATED	AT1G47480	
	Prupe.8G121500	0.976	1	Up_O1	CARBOXYLESTERASE 12-RELATED	AT3G48700	
***Hormones Metabolism/Signaling***	Prupe.6G295900	0.217	0	Up_O1	RECEPTOR-LIKE PROTEIN KINASE FERONIA	AT3G51550	
	Prupe.6G065600	1	7	Up_O1	BR-signaling kinase (BSK)	AT5G59010	
	Prupe.8G250300	0.5	1	Up_O1	BRI1 suppressor 1 (BSU1)-like 1	AT4G03080	
***Lactones Biosynthesis***	Prupe.7G162300	0.998	2	Up_O1	3-hydroxyacyl-CoA dehydrogenase	AT3G51000	YES
***Lipids Metabolism***	Prupe.4G132600	0.994	1	Qualit_O1	2,4-dienoyl-CoA reductase (NADPH) / 4-enoyl-CoA reductase (NADPH)	AT3G12800	
	Prupe.7G175400	0.989	0	Qualit_O1	PHOSPHOLIPASE D DELTA	AT4G35790	
	Prupe.8G162500	0.5	1	Up_O1	Phosphatidylinositol diacylglycerol-lyase / Phosphatidylinositol phospholipase C	AT4G38690	
	Prupe.3G064800	1	3	Qualit_O1	Peroxyureidoacrylate/ureidoacrylate amidohydrolase	AT3G16190	
	Prupe.6G180800	0.985	1	Up_O1	3-hydroxyacyl-[acyl-carrier-protein] dehydratase / D-3-hydroxyoctanoyl-[acyl carrier protein] dehydratase	AT5G10160	
	Prupe.5G079200	0.998	2	Qualit_O1	ACID CLUSTER PROTEIN 33	AT4G12230	YES
	Prupe.1G512000	0.104	0	Up_O1	Acetyl-CoA carboxylase 1	AT1G36160	
	Prupe.3G176900	1	3	Qualit_O1	3-hydroxyisobutyryl-CoA hydrolase-like protein 5	AT1G06550	
***Lipids/Redox Metabolism***	Prupe.6G063600	1	5	Up_O1	neutral ceramidase (ASAH2)	AT1G07380	
***Microtubules Organization***	Prupe.6G088500	0.083	0	Qualit_O1	KINESIN MOTOR PROTEIN-RELATED PROTEIN-RELATED	AT3G45850	
	Prupe.1G083500	0.236	0	Up_O1	Protein MOR1	AT2G35630	
	Prupe.5G206800	0.998	2	Up_O1	PROTEIN SPIRAL1	AT1G26355	
***Organelles Morphogenesis***	Prupe.3G172600	1	2	Qualit_O1	peroxisomal and mitochondrial division factor 2	AT1G06530	
	Prupe.6G326600	1	4	Up_O1	Ras homolog gene family, member T1 (RHOT1, ARHT1)	AT5G27540	
***Nucleosides and Nucleotides Biosynthesis***	Prupe.3G304300	1	3	Up_O1	URIDINE KINASE	AT5G40870	
***Peroxisome organization***	Prupe.3G220500	0.5	1	Qualit_O1	Peroxisome biogenesis protein	AT3G21865	
***Phenylpropanoids Metabolism***	Prupe.2G319700	1	5	Qualit_O1	Caffeate O-methyltransferase	AT5G54160	
	Prupe.2G263900	1	4	Qualit_O1	CHALCONE--FLAVONONE ISOMERASE 3-RELATED	AT5G05270	
	Prupe.3G194000	1	2	Qualit_O1	Cinnamyl alcohol dehydrogenase	AT1G72680	
***Photoperiodic Flowering Regulation***	Prupe.6G149500	0.614	1	Qualit_O1	Ubiquitin carboxyl-terminal hydrolase 12	AT5G06600	
***Polyamines metabolism***	Prupe.5G078900	0.977	1	Qualit_O1	AMINE OXIDASE-RELATED	AT4G12290	
	Prupe.1G255300	0.5	1	Up_O1	spermidine synthase (speE, SRM)	AT1G23820	
***Processing of Vacuolar Seed Protein Precursors***	Prupe.5G076300	0.998	2	Up_O1	HEMOGLOBINASE FAMILY MEMBER	AT1G62710	
***Protein Degradation***	Prupe.8G251000	0.998	1	Qualit_O1	nuclear protein localization protein 4 homolog (NPLOC4, NPL4)	AT2G47970	
***Protein Folding***	Prupe.8G161100	1	9	Up_O1	PEPTIDYL-PROLYL CIS-TRANS ISOMERASE	AT2G16600	
***Protein Modification***	Prupe.2G212600	0.621	1	Up_O1	oligosaccharyltransferase complex subunit alpha (ribophorin I) (OST1, RPN1)	AT2G01720	
***Protein Synthesis***	Prupe.1G133900	1	6	Qualit_O1	Eukaryotic translation initiation factor 2 subunit 3	AT1G04170	
	Prupe.1G177100	0.999	2	Qualit_O1	Eukaryotic translation initiation factor 3 subunit H	AT1G10840	
	Prupe.3G187100	1	5	Up_O1	60S ribosomal protein L27-3	AT4G15000	
	Prupe.3G286600	0.998	2	Up_O1	Large subunit ribosomal protein L7/L12 (RP-L7, MRPL12, rplL)	AT3G27830	
	Prupe.7G052700	1	7	Up_O1	Eukaryotic translation initiation factor 3 subunit E	AT3G57290	
***Protein Targeting***	Prupe.3G089600	1	1	Up_O1	SIGNAL RECOGNITION PARTICLE 54 KDA PROTEIN	AT1G48900	
***Pyrophosphate Metabolism and Photosynthate Partitioning***	Prupe.3G091900	0.998	1	Qualit_O1	Inorganic pyrophosphatase (ppa)	AT1G15690	
***Redox Metabolism***	Prupe.2G051700	1	3	Qualit_O1	RING FINGER PROTEIN 41, 151	AT3G54360	
***Regulation of L-ascorbic acid Biosynthetic Process***	Prupe.5G179900	0.998	2	Qualit_O1	Mannose-1-phosphate guanyltransferase alpha	AT1G74910	
***Regulation of Plant Cytokinesis/Abiotic Stress Response***	Prupe.5G105500	0.997	1	Up_O1	MITOGEN-ACTIVATED PROTEIN KINASE 5	AT4G01370	
***Regulation of Translation***	Prupe.6G154800	0.054	0	Qualit_O1	Protein argonaute 4	AT2G27040	
***RNA biogenesis***	Prupe.2G121400	0.998	2	Up_O1	DNA-directed RNA polymerases II and IV subunit 5A	AT3G22320	
***RNA biogenesis/Abiotic Stress Response***	Prupe.1G346100	1	3	Qualit_O1	ATP-dependent RNA helicase (EIF4A3, FAL1)	AT3G19760	
***RNA Splicing***	Prupe.3G036300	0.135	0	Qualit_O1	116 kDa U5 small nuclear ribonucleoprotein component (EFTUD2)	AT1G06220	
	Prupe.2G275100	0.564	0	Up_O1	SPLICING FACTOR 1	AT5G51300	
***Scaffolds in Cellular Signaling and Trafficking***	Prupe.5G125200	0.772	1	Qualit_O1	KINESIN LIGHT CHAIN	AT4G10840	
***Solute Transport***	Prupe.1G460700	0.563	1	Qualit_O1	Plasma membrane ATPase 4	AT2G24520	
***Specialized Metabolsim***	Prupe.6G325100	1	4	Up_O1	Aryldialkylphosphatase / Phosphotriesterase	AT3G05350	YES
	Prupe.3G026100	1	2	Up_O1	Cycloartenol synthase / 2,3-epoxysqualene--cycloartenol cyclase	AT2G07050	
	Prupe.4G002700	1	2	Up_O1	Farnesyl pyrophosphate synthase 2	AT5G47770	
	Prupe.2G160700	0.999	2	Up_O1	(+)-neomenthol dehydrogenase	AT3G61220	
***Telomerase biogenesis***	Prupe.6G137000	0.998	1	Up_O1	RuvB-like protein 1	AT5G22330	
***Vacuolar Protein Sorting***	Prupe.1G453100	0.5	1	Up_O1	AP-4 COMPLEX SUBUNIT BETA-1	AT5G11490	
	Prupe.2G056200	1	4	Up_O1	AP-4 complex subunit epsilon-1 (AP4E1)	AT1G31730	
	Prupe.1G365900	0.39	0	Up_O1	AP-2 complex subunit sigma-1 (AP2S1)	AT1G47830	
***Vesicles Trafficking***	Prupe.1G181200	0.285	0	Qualit_O1	Ras-related protein Rab-11A (RAB11A)	AT1G05810	
	Prupe.1G486400	0.27	0	Qualit_O1	TRAFFICKING PROTEIN PARTICLE COMPLEX SUBUNIT 9	AT5G11040	
	Prupe.8G013500	0.998	2	Up_O1	Ras-related protein Rab-18 (RAB18) // protein phosphatase 1L [EC:3.1.3.16] (PPM1L, PP2CE)	AT1G43890	
	Prupe.3G293600	0.53	1	Up_O1	Golgin candidate 6	AT3G27530	
	Prupe.2G271800	0.769	1	Up_O1	RAS-RELATED PROTEIN RABD2B-RELATED	AT5G47200	
	Prupe.1G555300	1	6	Up_O1	PATELLIN-3-RELATED	AT1G72160	
	Prupe.8G192300	1	1	Up_O1	Ras-related protein Rab-11A (RAB11A)	AT3G07410	
	Prupe.6G024700	0.999	2	Up_O1	Transmembrane emp24 domain-containing protein p24beta2	AT3G07680	
**Vesicles Trafficking/Redox Metabolism**	Prupe.6G134600	1	2	Up_O1	VESICLE-ASSOCIATED MEMBRANE PROTEIN 714	AT5G22360	
**Upregulated O2**							
***Abiotic Stress***	Prupe.3G078100	0.998	2	Up_O2	Chaperone protein DnaJ	AT2G22360	
	Prupe.2G178200	1	4	Up_O2	DESICCATION-RELATED PROTEIN LEA14-RELATED	AT1G01470	
	Prupe.7G125900	0.769	1	Up_O2	Aquaporin TIP1-1	AT2G36830	
***Anthocyanin transport***	Prupe.4G111700	0.998	2	Up_O2	GLUTATHIONE S-TRANSFERASE OMEGA-LIKE 1-RELATED	AT4G19880	
***Carbohydrates Metabolism***	Prupe.6G210900	0.414	0	Up_O2	phosphoenolpyruvate carboxykinase (ATP) (E4.1.1.49, pckA)	AT4G37870	
***Carbohydrates/Energy Metabolism***	Prupe.1G105800	0.118	0	Qualit_O2	[Pyruvate dehydrogenase (acetyl-transferring)] kinase / Pyruvate dehydrogenase kinase (phosphorylating)	AT3G06483	YES
***Cargo Delivery into the Peroxisome***	Prupe.6G036900	1	3	Qualit_O2	peroxin-5 (PEX5, PXR1)	AT5G56290	
***Carotenoid Biosynthetic Process***	Prupe.6G340000	1	10	Up_O2	Zeta-carotene desaturase, chloroplastic/chromoplastic	AT3G04870	
***Cell Wall Metabolism***	Prupe.4G262200	0.598	1	Qualit_O2	Polygalacturonase (PpPG22)	AT3G59850	
	Prupe.4G261900	0.747	1	Up_O2	Polygalacturonase (PpPG21)	AT3G59850	
	Prupe.2G300900	0.99	1	Qualit_O2	Probable polygalacturonase	AT3G48950	
	Prupe.3G050200	1	3	Up_O2	BETA-GALACTOSIDASE 1	AT3G13750	
	Prupe.5G131300	0.977	1	Up_O2	ENDO-1,4-BETA-GLUCANASE PpEG1	AT1G02800	
	Prupe.6G075100	0.995	1	Up_O2	Expansin 3 (PpExp3)	AT2G39700	
	Prupe.4G157600	0.474	0	Up_O2	PROTEIN TRICHOME BIREFRINGENCE-LIKE 41	AT3G14850	
	Prupe.1G114500	0.745	1	Up_O2	Plant invertase/pectin methylesterase inhibitor (PMEI)	AT1G47960	
***Chloroplast Division***	Prupe.4G013700	0.5	1	Up_O2	Tetratricopeptide repeat protein 1	AT1G62390	
***Endocytosis***	Prupe.6G275500	0.998	2	Up_O2	CLATHRIN LIGHT CHAIN 2	AT3G51890	
***Energy Metabolism***	Prupe.6G244100	1	3	Up_O2	ADENYLATE KINASE 1, MITOCHONDRIAL-RELATED	AT2G37250	
	Prupe.3G092100	0.998	2	Up_O2	NADH dehydrogenase [ubiquinone] flavoprotein 2, mitochondrial	AT4G02580	
	Prupe.6G249900	1	7	Up_O2	INORGANIC PYROPHOSPHATASE-LIKE PROTEIN	AT3G53620	
***Hormones Metabolism***	Prupe.3G209900	0.643	1	Up_O2	1-aminocyclopropane-1-carboxylate oxidase (ACO1)	AT1G05010	
	Prupe.1G111900	0.996	1	Up_O2	GIBBERELLIN 2-BETA-DIOXYGENASE 4	AT1G02400	YES
	Prupe.7G188800	1	2	Up_O2	2-OXOGLUTARATE (2OG) AND FE(II)-DEPENDENT OXYGENASE-LIKE PROTEIN	AT1G14130	
***Lactones biosynthesis***	Prupe.6G192200	0.244	0	Qualit_O2	SERINE/THREONINE-PROTEIN KINASE SRK2C	AT1G78290	YES
***Lipids Metabolism***	Prupe.7G076500	0.998	2	Qualit_O2	Omega-6 fatty acid desaturase, endoplasmic reticulum isozyme 2	AT3G12120	YES
***Lipids/Hormones Metabolism***	Prupe.1G332000	0.769	1	Qualit_O2	Probable peroxygenase 4	AT1G70670	YES
***Lipids Metabolism/Signaling***	Prupe.4G089300	1	2	Up_O2	GLYCOLIPID TRANSFER PROTEIN-RELATED	AT2G34690	
***MicroRNAs processing***	Prupe.6G153800	0.5	1	Up_O2	ARSENITE-RESISTANCE PROTEIN 2	AT2G27100	
***Mitotic Cell Cycle Progression***	Prupe.1G154700	0.503	1	Up_O2	RAN GTPase-activating protein 2	AT5G19320	
***Nuclear Morphology and Heterochromatin Organization***	Prupe.6G214100	0.621	1	Qualit_O2	Putative nuclear matrix constituent protein 1-like protein	AT5G65770	
***Protein Degradation/Signaling***	Prupe.1G040200	0.383	0	Qualit_O2	E3 UBIQUITIN-PROTEIN LIGASE XBAT35-RELATED	AT3G23280	
	Prupe.7G170300	1	4	Up_O2	26S proteasome regulatory subunit N10 (PSMD4, RPN10)	AT4G38630	
***Protein Folding***	Prupe.1G348800	0.745	1	Up_O2	prefoldin alpha subunit (pfdA, PFDN5)	AT5G23290	
	Prupe.1G118100	0.655	0	Up_O2	Peptidyl-prolyl cis-trans isomerase CYP21-3, mitochondrial	AT3G66654	
***Protein Synthesis***	Prupe.7G265100	1	11	Up_O2	EUKARYOTIC TRANSLATION INITIATION FACTOR 4 GAMMA	AT5G57870	
	Prupe.1G274500	0.998	2	Up_O2	Large subunit ribosomal protein L34e	AT1G26880	
	Prupe.5G147500	1	4	Up_O2	Large subunit ribosomal protein L7Ae (RP-L7Ae, RPL7A)	AT3G62870	
	Prupe.6G192100	0.564	1	Up_O2	Small subunit ribosomal protein S6e (RP-S6e, RPS6)	AT5G10360	
	Prupe.7G268100	1	3	Up_O2	Small subunit ribosomal protein S24e (RP-S24e, RPS24)	AT5G28060	
***Protein Targeting***	Prupe.7G179600	0.227	0	Up_O2	PROTEIN GLUTAMINE DUMPER 7	AT5G66030	
	Prupe.1G362200	1	5	Up_O2	Early endosome antigen 1	AT1G20110	
***Protein Transport***	Prupe.5G110100	0.998	2	Up_O2	RETICULON-LIKE PROTEIN B4	AT4G11220	
***Regulation of Transcription***	Prupe.1G054700	0.5	1	Qualit_O2	STRUCTURAL MAINTENANCE OF CHROMOSOMES SMC FAMILY MEMBER	AT3G23980	
	Prupe.8G189400	0.368	0	Up_O2	DnaJ homolog subfamily C member 2 (DNAJC2)	AT3G11450	
***Regulation of Translation***	Prupe.6G360600	0.567	0	Qualit_O2	PROTEIN PHOSPHATASE RELATED	AT3G02830	
***RNA Splicing***	Prupe.4G042600	0.101	0	Qualit_O2	splicing factor 3B subunit 2 (SF3B2, SAP145, CUS1)	AT4G21660	
	Prupe.7G153200	0.23	0	Up_O2	U1 small nuclear ribonucleoprotein 70kDa (SNRP70)	AT3G50670	
	Prupe.8G029000	0.5	1	Up_O2	ATP-dependent RNA helicase DDX23/PRP28 (DDX23, PRP28)	AT2G33730	
	Prupe.6G190500	0.536	0	Qualit_O2	SNW domain-containing protein 1 (SNW1, SKIIP, SKIP)	AT1G77180	
	Prupe.6G349400	0.564	1	Up_O2	Cell division cycle 5-like protein	AT1G09770	
***RNA-mediated Post-transcriptional Gene Silencing***	Prupe.6G115400	0.395	0	Up_O2	Protein argonaute 5	AT2G27880	
***Senescence***	Prupe.7G173400	0.5	1	Up_O2	LA-RELATED PROTEIN 1B-RELATED	AT4G35890	
***Signaling***	Prupe.4G021300	0.571	1	Up_O2	CALCIUM-DEPENDENT PROTEIN KINASE 29	AT4G04720	
	Prupe.8G057600	0.567	1	Up_O2	SERINE/THREONINE-PROTEIN KINASE	AT4G09570	
	Prupe.2G134200	0.998	2	Up_O2	Nuclear pore glycoprotein p62	AT2G45000	
***Sugar Transport***	Prupe.8G101200	0.998	2	Up_O2	POLYOL TRANSPORTER 5 PePOL5	AT3G18830	
***Specialized Metabolsim***	Prupe.5G106300	0.772	1	Up_O2	2-C-methyl-D-erythritol 2,4-cyclodiphosphate synthase / MECDP-synthase	AT1G63970	
	Prupe.8G032700	0.607	1	Up_O2	Sterol 3-beta-glucosyltransferase	AT1G43620	
	Prupe.3G097700	0.5	1	Up_O2	cinnamoyl-CoA reductase (CCR)	AT1G15950	
***Vacuolar Sorting***	Prupe.7G171800	0.5	1	Up_O2	VACUOLAR SORTING PROTEIN 35	AT2G17790	
	Prupe.8G143700	0.607	1	Up_O2	Vacuolar-sorting receptor 3	AT2G14740	

^a^*Qualit* Qualitative change in expression at a given condition, *Up* Upregulated (quantitative change) at a given condition

^b^According to Sánchez et al. An integrative "omics" approach identifies new candidate genes to impact aromavolatiles in peach fruit. BMC Genomics. 2013 May 23;14:343; Li et al. Identification of volatile and softening-related genes using digital gene expression profiles in melting peach. Tree genetics & genomes 11.4 (2015): 71

**Fig. 4:**
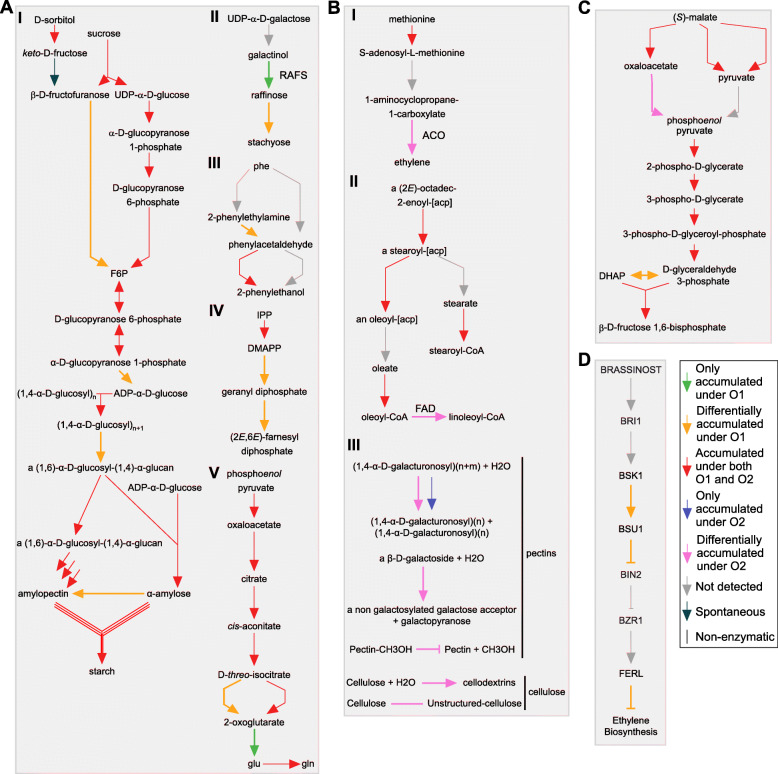
Analysis of biological processes and pathways associated to differentially accumulated proteins. **a** Sucrose and sorbitol conversion into fructose 6-phosphate (F6P), and thereafter into starch, would preferentially occur at the mature fruit stage (panel I, MetaCyc pathways PWY-3801 and PWY-622). Raffinose and stachyose (PWY-5337), phenylacetaldehyde (PWY-5751), farnesyl diphosphate (PWY-5123) and glutamine (PWY-6549) biosynthetic pathways also had proteins that were more abundant in mature fruit (panels II to V). **b** In turn, ethylene (ETHYL-PWY) and linoleoyl-CoA (PWY-6001) would be preferentially synthesized in ripe fruit due to the preferential accumulation of enzymes related to these pathways at this stage (panels I and II). Cell-wall dismantling would also be favored when the fruit ripens due to the accumulation of pectin and cellulose modifying proteins and enzymes (panel III). **c** Gluconeogenesis (GLUCONEO-PWY) related enzymes were found accumulated in mature and ripe fruit, but with differences at the glycerone phosphate - D-glyceraldehyde 3-phosphate interconversion step (O1, EC 5.3.1.1) and the oxaloacetate conversion into phosphoenolpyruvate (O2, EC 4.1.1.49), which had differentially accumulated proteins associated to mature and ripe fruit. **d** Ethylene biosynthesis in mature fruit would be negatively modulated by the action of the signaling cascade that would begin with the action of brassinosteroids and culminate with Feronia-like receptor kinases (FERLs) action upon genes involved in ethylene biosynthesis. F-6-P - fructose 6-phosphate; RAFS - raffinose synthase; phe - phenylalanine; IPP - isopentenyl diphosphate; DMAPP - prenyl diphosphate; glu - glutamate; gln - glutamine; ACO - 1-aminocyclopropane-1-carboxylate oxidase; FAD - Fatty acid desaturase

Among the most relevant pathways associated to proteins with differential accumulation in mature fruit, there were several that could have a direct impact in the fruit organoleptic quality: Sucrose and sorbitol conversion into fructose 6-phosphate (PWY-3801), raffinose and stachyose (PWY-5337), phenylacetaldehyde (PWY-5751), farnesyl diphosphate (PWY-5123) and glutamine (PWY-6549) biosynthesis (Fig. 4a, panels I to V). In ripe fruit, we selected the ethylene (ETHYL-PWY) and linoleoyl-CoA (PWY-6001) biosynthesis pathways (Fig. 4b, panels I and II). Proteins related to cell wall disassembly were also differentially accumulated in ripe fruit (Fig. 4**b**, panel III). Transport reactions, such as sorbitol transport, would also be upregulated in ripe fruit (Prupe.8G101200, Table 1).

Almost all steps of the gluconeogenesis pathway (GLUCONEO-PWY) were represented by proteins characterized in this study (Fig. 4**c**), indicating that the pathway was active in both stages. There were very few differences in the accumulation of the proteins between mature and ripe fruit. However, subtle differences were found including the step that interconverts dihydroxyacetone phosphate (DHAP) into D-glyceraldehyde 3-phosphate (EC 5.3.1.1) and the step that converts oxaloacetate into phosphoenolpyruvate (EC 4.1.1.49), with higher accumulation levels in mature and ripe fruit, respectively.

In terms of regulatory pathways, the signaling cascade that leads to the ethylene biosynthesis inhibition through the action of brassinosteroids over kinases such as the serine/threonine-protein kinase BRASSINOSTEROID-SIGNALING KINASE (BSK, Prupe.6G065600) and the receptor-like protein kinase FERONIA (Prupe.6G295900) was well represented in our mature fruit dataset (Fig. 4d).

### Potential biomarkers of peach fruit ripening include cell wall modifying proteins and proteins involved in plant hormone biosynthesis

**Fig. 5:**
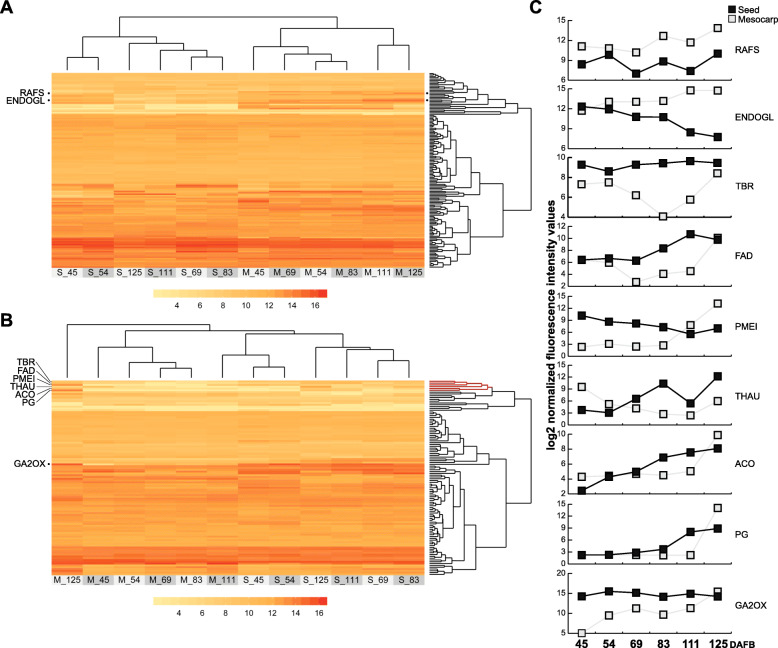
Transcriptome analysis of protein-coding genes assessed in this work throughout the fruit development curve. **a** Gene expression data (GEO GSE71561) from the genes that code for the 248 differentially accumulated proteins assessed in this work, was retrieved from the Fantasy variety for six developmental stages and two tissues, seeds and mesocarp. Hierarchical cluster analysis revealed that, when considering the 140 genes from differentially accumulated proteins in mature fruit mesocarp, stages 111 and 125 DAFB (M_111, M_125) were set apart from all other conditions. **b** This pattern changed when considering proteins that were differentially accumulated in ripe fruit, with stage M_125 being set apart from any other cluster. **c** Expression patterns of the genes that showed their highest expression at M_125, highlighted at the left side of the hierarchical clusters shown in (**a**) and (**b**), is depicted. Several of these genes were concentrated in the same cluster (highlighted in red in the upper segment of the figure (**b**)). RAFS - raffinose synthase, Prupe.6G032400; ENDOGL - endoglucanase - Prupe.5G118000; TBR - Protein Trichome Birefringence-like 41, Prupe.4G157600; FAD - Fatty acid desaturase - Prupe.7G076500; PMEI - Plant invertase/pectin methylesterase inhibitor, Prupe.1G114500; THAU - thaumatin, Prupe.3G144100; ACO - 1-aminocyclopropane-1-carboxylate oxidase, Prupe.3G209900; PG - polygalacturonase, Prupe.4G262200; GA2OX - Gibbellerin 2-beta-dioxygenase 4, Prupe.1G111900

In order to determine which protein-coding genes could be used as biomarkers to differentiate between mature and ripe fruit stages, gene expression data across six developmental stages in fruit seeds and mesocarp, was plotted as heatmaps (Fig. 5, Supplementary Fig. 3A). An optimal biomarker would be a gene whose expression is low at any stage and tissue, but that achieves maximum expression in the ripe fruit mesocarp tissue. When considering protein-coding genes from mature fruit (Fig. 5a), only two proteins came close to displaying this pattern, a raffinose synthase (RAFS, Prupe.6G032400) and an endoglucanase (ENDOGL, Prupe.5G118000, Fig. 5c). When considering protein-coding genes from ripe fruit (Fig. 5b), this pattern was mainly displayed by seven protein-coding genes, six of which were clustered. These six clustered genes included three cell wall modifying proteins (polygalacturonase, Prupe.4G262200; pectin methylesterase inhibitor, Prupe.1G114500; and protein trichome birefringence-like 41, Prupe.4G157600), the main peach fruit 1-aminocyclopropane-1-carboxylate oxidase (Prupe.3G209900), an omega-6 fatty acid desaturase (Prupe.7G076500), and a thaumatin (Prupe.3G144100). The seventh protein was a gibberellin 2-beta-dioxygenase (Prupe.1G111900). Interestingly, several of these proteins were consistently found to be more abundant in mesocarp of O’Henry ripe fruit using 2D-gel electrophoresis (Supplementary Table 1, Supplementary Fig. 4) [27]. It is also interesting to note that, based on gene expression clusters, the “developmental stage” would be more relevant in terms of coordinating gene expression than “tissue specificity” (Supplementary Fig. 3A).

### Protein-coding genes involved in fruit ripening are potential targets for transcriptional factors enriched in NAC and SANT/Myb domains

**Fig. 6:**
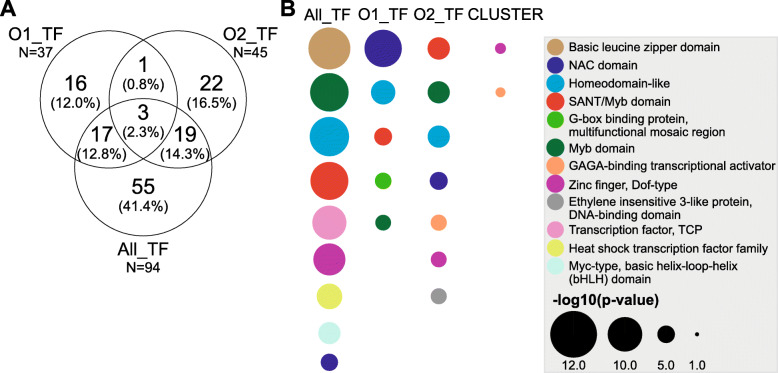
Analysis of transcription factors with over-represented targets in the set of differentially accumulated proteins. **a** Ninety-four, thirty-seven and forty-five transcription factors (TFs) were found to have over-represented targets among the proteins assessed in this study (“ALL_TF”), those differentially accumulated in mature fruit (“O1_TF”), and those differentially accumulated in ripe fruit (“O2_TF”).”). **b** The only TF that targeted differentially accumulated proteins in mature and ripe fruits in this study (purple labeled) was the protein Prupe.7G1499700, which displayed an accumulation pattern closely related to the one from the ACO1 (Prupe.3G209900) across the fruits developmental and ripening stages. **c** An analysis of TF protein domains indicated that “ALL_TF”, “O1_TF” and “O2_TF” were composed of TFs with different architecture. The analysis was also extended to those TFs that targeted the seven protein-coding genes with high expression in ripe fruit (“Cluster”, Fig. 4, panel **(c)**. Time point’s labels in **(b)** are the same as in Fig. 5

The analysis of transcription factors (TFs) that had over-represented targets in the set of genes that coded for differentially accumulated proteins in mature and ripe fruit indicated that two different families of TFs would regulate genes expressed at mature and ripe fruit. Thus, 94, 37 and 45 TFs were found to possess over-represented targets among the 1663 protein-coding genes assessed in this study, among those differentially accumulated in mature fruit and among those differentially accumulated in ripe fruit, respectively (Fig. 6a). The TF that targeted protein-coding genes for both mature and ripe fruit, a homeobox-leucine zipper protein ATHB-40 (Prupe.7G149700), displayed an expression profile that correlates with the ACO1 gene, i.e. a high expression peak at the stage of fruit ripening (Fig. 6b). An analysis of the TF protein domains indicated that these three sets of TFs have different domain composition (Fig. 6c), with genes coding for proteins more accumulated in mature fruit displaying mainly an enrichment as targets of NAC domain TFs whereas genes from proteins more accumulated in ripe fruit being enriched as targets of MYB domain TFs.

## Discussion

Proteomic approaches based on 1D electrophoresis followed by MS (Fig. 1) have been successfully used to better understand fruit ripening in Rosaceae family species such as apricot [33] and strawberry [34]. When compared to proteins extracted from ripening *P. armeniaca* using 1D SDS-PAGE gel followed by MS, 76% of the 245 proteins identified in mature/ripe apricot fruit were also identified in mature/ripe peach fruit [33]. Moreover when compared to other three *P. persica* species gel-based proteomic studies which published all the proteins characterized (Supplementary Table 3), on average an 85% of the characterized proteins matched the ones found in our study. Adding the fact that the physicochemical properties of *P. persica* primary transcripts’ proteome were similar to those of the proteome analyzed in the present study (Supplementary Table 2, Supplementary Fig. 2) and that low abundant proteins, such as TFs, could be detected in our dataset, the current analysis seems to truly reflect the fruit proteome, but with a bias in terms of primarily representing proteins with average physicochemical properties (Supplementary Fig. 2). Comparison with iTRAQ proteome datasets would be highly informative, however only differentially accumulated proteins are reported in these publications with emphasis on the effect of postharvest treatments such as cold storage or disease incidence in ripening process [35–39] (Supplementary Table 3).

In order to take full advantage of the proteins characterized in systems such as ripe fruit, it is important to follow a robust pipeline of MS analysis and protein quantification, to increase the confidence in the identifications performed and to support differential accumulation analysis. MS analysis was performed using a stringent cutoff for both peptide and protein match. Next, protein quantification was performed using the average total ion chromatograms (Average TIC) [40] followed by pre-treatment data to get a data distribution as close to normal as possible (Supplementary Fig. 1), data scaling and centering, and data cleaning, to keep only those proteins that had a proper number of replicates to perform a statistical analysis of differential accumulation. All these steps explain the drop from 2740 detected proteins to 1663 assessed proteins. It is also worth to remember that label-free quantification, such as the one used in this work, is a good choice for proteomic analysis that seeks to characterize as many unbiased proteins as possible. However, variations are higher than in techniques that rely on labeling, since in label-free quantification samples are individually prepared and comparison occurs later, during data analysis [41]. In addition, variability among fruits in the field can be very high, even if the fruits were manually selected based on size, color and physicochemical parameters. Therefore, in many cases a protein may vary widely between different conditions, and its abundance variation due to biological and technical issues might hurdle the ability to identify this protein as differentially accumulated.

The fruit mesocarp proteome identified in this work (1663 proteins) was functionally related to carboxylic acid metabolism, intracellular transport and protein folding (Fig. 2a). Transport proteins were mainly related to carbohydrates, ions, and vesicle-mediated transport (Supplementary Table 1), with very few transporters differentially accumulated. Protein folding, in turn, was mainly related to chloroplast chaperonins [42]. Both sets point to a fruit mesocarp with active cellular compartment transport and protein folding.

Mature and ripe fruit stages are the last in the fruit developmental curve, making it possible to compare protein-coding genes from our dataset with datasets that characterized ripening-related genes in *P. persica* (GEO dataset GSE71561) [32]. Protein-coding genes expressed in mature/ripe fruit seemed to be time and tissue-specific, given that close to 25% peak their expression levels in ripe fruit when compared to other developmental stages, and 52% peaked their expression in ripe fruit mesocarp, compared to other tissues (Fig. 2b and c). Fruit-specific genes that displayed the strongest differentiation between peach and almond (*P. dulcis*) were also the most highly expressed, pointing to a functional specialization of protein-coding genes that are highly expressed in fruit [43]. Our results point to the same behavior in functionally specialized fruit proteins, i.e., proteins with high accumulation in fruit could be particularly involved in its metabolism.

Samples and protein abundance differences between mature and ripe fruit mesocarp were striking, with a PCA first component splitting both samples and 14.9% of the assessed differentially accumulated proteins (Fig. 3A, Table 1). This reinforces the notion that mature and ripe fruits are different not only in terms of metabolic profiles, but also at the proteome level. Processes triggered in the mature fruit would be directed to glycogen and isocitrate metabolism, and protein localization (Fig. 3B), whereas a manual inspection indicated that protein-driven cell wall modification was much more represented in ripe fruit than in mature fruit (nine versus two proteins, Table 1, Fig. 4b). Seven of these cell wall proteins have been experimentally shown to be ethylene responsive: polygalacturonase (PG) Prupe.2G300900 [44], PpPG21 (Prupe.4G261900) [44], PpPG22 (Prupe.4G262200) [14, 44, 45], beta-galactosidase (Prupe.3G050200) [14], pectin methylesterase inhibitor (Prupe.1G114500) [46], expansin 3 (Prupe.6G075100) [45], and endo-1,4-beta-glucanase PpEG1 (Prupe.5G131300) [47], in according to the increase in ethylene production at ripe stage [28] highlighting its relevance as a ripening promoting hormone in melting peach fruit varieties. In addition, protein-coding genes from PpEG1, PpPG21, Prupe.2G300900 and Prupe.3G050200 (**PpBGAL2**) belonged to the same gene cluster with higher expression in ripe fruit compared to mature fruit, in the white flesh fast-melting peach “Hu Jing Mi Lu” (HJ) [48], pointing to a conserved mechanism of cell wall dismantling in peach fruits with a different genetic background. PpBGAL2 transcript was also shown to be highly correlated with the fruit softening in the melting variety XH8, with expressions 3–4 times higher during later storage compared to early storage [49].

Peach fruit size is controlled throughout its development mainly by cell divisions and enlargement of mesocarp cells [50]. These processes display a temporal control during the fruit development, with two stages of faster growth alternating with two stages of slower increases in fruit diameter, represented by a double-sigmoid growth pattern [50, 51]. Fruit size increase during the ripening process ceases, however how this size control is accomplished is not clear. One of the mechanisms that are involved in increasing the fruit cell size is the genome size amplification by endoreduplication [52], where endopolyploid cells arise from variations of the canonical cell cycle that replicate the genome without cell division. Recently a GUANYLATE-BINDING PROTEIN1 (SlGBP1) was characterized as a possible inhibitor of cell division in tomato. This protein would maintain endopolyploid cells in a non-proliferative state [53]. The best *P. persica* homologue of SlGBP1 (Prupe.4G053400, 70.6% identity) was found differentially accumulated in ripe fruit in this study, indicating that endopolyploid cells division would be inhibited during the fruit ripening, providing a molecular mechanism to explain the lack of fruit size increment in this developmental stage in peach.

### Key processes involved in the organoleptic changes that occur in the transition from mature to ripe fruit

The ripening process involves changes in the fruit mesocarp flavor, color, aroma, and texture that have a direct impact on the fruit organoleptic quality. By studying the proteomic changes that are triggered during ripening, it would be possible to underscore some of the critical molecular processes involved in this transition. During this research, proteins involved in hormone, soluble sugars, organic acids, lipids, specialized metabolism and vesicle mediated trafficking and protein transport were correlated with this transition, and their possible role in fruit ripening is discussed below.

### Hormone metabolism

There is multiple evidence that the plant hormone ethylene is involved in the regulation of the ripening process in climacteric fleshy fruit. Upon binding to its receptors, ethylene’s signal is propagated to several downstream components which in turn target promoters of many ethylene-inducible genes, directly involved in the dramatic changes that occur during the transition from mature to ripe fruit [54]. The ACC synthase (ACS) and ACC oxidase (ACO) enzymes are responsible for turning S-Adenosyl methionine (SAM) into ethylene. In peach, the abundance of the ACO isoform ACO1 (Prupe.3G209900) transcript and protein correlates very closely with fruit ripening [27, 55, 56]. In this work, ACO1 also displayed a similar pattern, being more abundant in ripe fruit than in mature fruit (Table 1, Fig. 3D), validating ethylene production measured on the mature and ripe fruit stages at the molecular level [28].

The possible signaling cascades that modulate ethylene biosynthesis in climacteric fruit are poorly understood. Recently, in apple (*Malus×domestica*) and tomato (*Solanum lycopersicum*), both climacteric fruits, Feronia-like receptor kinases (FERLs) were shown to act as negative regulators of fruit ripening by inhibiting ethylene production [57]. In the current study, a *P. persica* FERL (Prupe.6G295900), with over 75% of identity with the apple MdFERL1 and the tomato SlFERL1, was characterized as more abundant in mature than in ripe fruit (Table 1). In addition, upstream key regulators of FERL, such as BRASSINOSTEROID-SIGNALING KINASE (BSK) and BRI1 SUPPRESSOR (BSU) [58], were also more abundant in mature than in ripe fruit. This pattern suggests that this signaling pathway could regulate ethylene biosynthesis during peach fruit ripening, as its downregulation in the transition from mature to ripe fruit is correlated with the increase in ethylene biosynthesis in ripe fruit.

### Sugar metabolism

The sugar alcohol sorbitol is the main metabolite used to mobilize photosynthesis-derived carbohydrates from leaves to fruits in Rosaceae [59], being highly correlated with fruit taste and aroma [60], and of great interest for fruit breeders given its nutritional and sweetener qualities [61]. At a molecular level, sorbitol modulation could impact fruit quality by affecting sugar-acid balance and starch accumulation [62]. How this effect is generated is not clear, but it is postulated that sorbitol is catabolized in the cytosol, being the main driver of structural compounds biosynthesis and respiration in the peach fruit [19]. In mature fruit, sorbitol could be used to generate fructose, which in turn could be metabolized into fructose 6-phosphate (F6P), by the enzyme fructokinase (Prupe.1G196700), whose abundance is high at this stage (Fig. 4b). F6P can be used to synthesize sucrose, the predominant soluble sugar in mature fruit, or enter the glycolytic pathway [60]. A decrease in the accumulation of fructokinase in ripe fruits indicated an enhanced conversion of sucrose to fructose via suppression of sucrose synthesis and fructose phosphorylation. Similar results were observed in other peach cultivar [37], where a decrease in different isoforms of both hexokinase (ppa004610m/Prupe.1G444000) and fructokinase (ppa007069m/Prupe.3G160500) accumulation were observed in fruits ripening and senescence at room temperatures. In ripe fruit, the putative sorbitol transporter (SOT) (Prupe.8G101200) accumulated more than in mature fruit (Table 1). This protein is a close homologue to candidate SOTs in pear (PbSOT19/21) [63], apple (SOT6) [64] and sour cherry (*P. cerasus*, PcSOT1) [65]. SOTs are represented by an expanded gene family in apple and peach [66], which is made up of 16 genes in *P. persica*. The ones that display the highest expression at the ripening stage were Prupe.8G101200 (PpePOL5) [67] and Prupe.3G071100 (Supplementary Fig. 3B). Thus, the PpePOL5 transporter could be partially responsible for an increase in sorbitol content in ripe fruit of the O’Henry variety, compared to mature fruit [68].

An intriguing question still under debate is about the role of stored malate in fruit metabolism during the ripening process, as malate synthesis and not dissimilation is detected throughout ripening [17]. Malate levels are mainly regulated by its synthesis due to cytosolic carboxylation of phosphoenolpyruvate (PEP), its degradation due to decarboxylation also in the cytosol or by the conversion of tri- and dicarboxylates in the mitochondria, glyoxysome or cytosol [69]. Malate can be used as a sugar precursor through gluconeogenesis, to help regulate nitrogen metabolism [17], as well as valves to balance metabolic fluxes given their function in indirect transport of reducing equivalents [70]. According to the proteomic profiles assessed in this study, malate would be catabolized by the gluconeogenesis process to different metabolites in mature and ripe fruit (Fig. 4). In mature fruit, the higher abundance of the protein triosephosphate isomerase would point to a preferred catalysis of malate to glyceraldehyde 3-phosphate, a triose phosphate that can be oxidized into pyruvate, providing the mature fruit with ATP and NADH. In ripe fruit, the higher abundance of the enzyme phosphoenolpyruvate carboxykinase would help to keep the appropriate physicochemical conditions for the use of glutamate as nitrogen source for the biosynthesis of amino acids required at this stage [17]. Our results also indicated a decrease in other organic acids such as citrate. The enzyme pyruvate dehydrogenase kinase (PDK) inactivates the pyruvate dehydrogenase (PDH). PDK (Prupe.1G105800) was detected only in ripe fruits, thus we could argue that in ripe fruits PDH is inhibited, which could decrease the synthesis of acetyl-CoA an consequently the Krebs cycle organic acids. Our results are in agreement with very recent data by Zheng et al. [71] showing that in low acid peach cultivars such as O’Henry, PDK expression is increased in comparison to high acid peach cultivars, which would impact the citrate synthesis pathway and its accumulation.

Among the soluble sugars present in the peach fruit, sucrose, fructose and glucose do not change much during the transition from mature to ripe fruit; in contrast to xylose, fucose and raffinose, according to an assessment of 15 *P. persica* varieties [2]. In the current analysis, two key enzymes involved in raffinose biosynthesis (raffinose and stachyose synthases, Table 1, Fig. 4a) were more accumulated during the mature stage, indicating that in the O’Henry variety this metabolite accumulation would occur at the mature stage. Raffinose is an important metabolite given its antioxidant properties and role in stress tolerance [2].

Starch biosynthesis and accumulation is believed to happen at the early stages of fruit development, followed by its consumption until almost undetectable levels at maturity [60]. However, enzymes involved on starch biosynthesis, such as 1,4-alpha-glucan-branching enzyme (SBE, Prupe.1G354000) and glucose-1-phosphate adenylyltransferase small subunit (APS1/AGP, Prupe.3G192600) were still detected in mature fruit, with a drop in their levels in ripe fruit (Fig. 3C). This same APS1/AGP was detected by iTraq proteome and was showed to decrease in fruits ripening at low temperatures [37]. SBE was also detected by others in the mesocarp of ripe peach fruits [72], indicating that starch biosynthesis could still be active at later stages of fruit development. Another interesting fact is the increased accumulation of the atypical cysteine/histidine-rich thioredoxin 4 *P. persica* homologous (ACHT4, Prupe.7G001600, 64% identical) in ripe fruit. ACHT4 has been characterized in *A. thaliana* as a molecular switch of APS1, being able to quench APS1 activity [73]. By combining decreased accumulation of SBE and APS1 with an increase of ACHT4, the ripe fruit metabolism could downregulate starch content at this final developmental stage.

### Fruit aroma and lipid metabolism

Among the many dozens of volatiles peach fruits can produce, the γ- and δ-decalactones, C6 aldehydes and alcohols, terpenoids and volatile esters are the ones that are mainly related to fruit aroma [8, 74, 75]. Volatile esters are the product of alcohol acyltransferase-mediated biosynthesis, and degradation through carboxylesterase (CXE) activity [75]. In tomato, low ester levels, which positively correlate with fruit liking, were associated to the enzyme SlCXE1 [76]. In peach, the carboxylesterase PpCXE1 (Prupe.8G121900) was shown to be able to degrade volatile acetate esters [75]. In the present study, two CXE proteins were found to be more abundant in mature fruit than in ripe fruit, Prupe.1G439300 and Prupe.8G121500. The genes coding for these proteins were top-7 and top-8 in expression level in ripe fruit, among 19 putative CXEs in *P. persica* [75]. Prupe.1G439300 is the best homologue to the tomato SlCXE1 [75] and its protein-coding gene displayed a peak of expression in ripe fruit (GEO dataset GSE71561), being a good candidate for further characterization.

Fruits can synthesize lactones that attract feeders for seed dispersal, mostly the γ-lactones decano-4-lactone and/or dodecano-4-lactone [77]. These compounds, which primarily derive from oleic acids or derivatives, are metabolized by a series of enzymes, including epoxide hydrolases, to generate lactones such as undecano-4-lactone [77]. They can be found both in fruit skin as well as mesocarp [74]. According to our data, lactone metabolism would also be differentially regulated in mature and ripe fruit, due to preferential accumulation of epoxide hydrolase EPH2 (Prupe.7G162300) [78] in mature fruit. On the other hand, the SRK2C kinase (Prupe.6G192200), which has been correlated to lactones using QTL analysis [79], is exclusively found in ripe fruit (Table 1). The target of SRK2 is unknown, and given its high expression (GEO dataset GSE71561), differential accumulation during the ripening process (Table 1), and correlation with lactone biosynthesis, it becomes an interesting candidate for further characterization.

Fruit ripening has been characterized as a senescing process, with cell membrane deterioration being the hallmark. Phospholipase D (PLD) is among the most relevant proteins involved in cell membrane deterioration in ripening fruits, acting upon phospholipids to generate phosphatidic acid (PA) and a free head group [80]. PLD action is also able to trigger a myriad of cellular processes, and therefore its activity is tightly regulated [81]. PLDs have been grouped into five classes (α, β, γ, δ and ζ) according to several parameters such as domain structure and biochemical properties. Similar to most plant PLDs, PLDδ requires Ca^2+^ and is stimulated by phosphatidylinositol 4,5-bisphosphate (PIP2) [81]. However, *Arabidopsis* PLDδ displays a distinctive property, which is to be activated by oleic acid [81]. A peach PLDδ (Prupe.7G17540) was found to be more abundant in mature fruit than in ripe fruit (Table 1).

Fatty acid desaturase 1 (FAD1, Prupe.7G076500), a ω-6 Oleate desaturase [8], displayed increased abundance in ripe fruit (Fig. 4b, panel II), a pattern similar to the one displayed by its transcript in the *P. persica* Yulu variety [74], in the GSE71561 dataset, and by its closest orthologue in *Fragaria vesca* [79]. FAD1 gene expression was also sensitive to the ethylene signal transduction pathway inhibitor 1-methylcyclopropene (1-MCP), a compound known to negatively affect peach fruit aroma [82]. This increase could imply that FAD1 could be involved in linoleic and linolenic acids biosynthesis in peach ripe fruit [8], or could be involved in γ-decalactone biosynthesis, as proposed for the *F. vesca* FaFAD1 [79].

### Solute transport

Aquaporins are transmembrane proteins that enable water and small neutral solute translocation across cellular membranes. Among the different types of aquaporins, those located in the vacuolar membranes are called tonoplast intrinsic proteins (TIPs). TIPs are classified into five groups (TIP1–5), and are able to transport hydrogen peroxide and glycerol, in addition to water [83]. The *P. persica* aquaporin TIP1–1 (Prupe.7G125900) was found to be more abundant in ripe than in mature fruit mesocarp (Table 1). A close homologous (93% identity) protein-coding gene from sweet cherry (*P. avium*) was among the three most expressed aquaporins in the fruit, being mainly expressed in mesocarp throughout fruit development [84]. The *P. persica* PpTIP1–1 transcriptional pattern was very similar (GEO dataset GSE71561), with the gene being expressed at a regular level throughout mesocarp development. This indicates that both Prunus TIP1–1 aquaporins conserved their tissue localization and expression patterns. Moreover, it indicates that these aquaporins could be key to transport water across the vacuole in the *Prunus* fruit mesocarp [84].

### Vesicle mediated trafficking and protein transport

Protein trafficking, mediated by vesicular transport, was also shown to be a very important process during the fruit ripening transition (Figs. 2 and 3). Vesicle transport can be divided in three broad consecutive steps: vesicle budding from a donor membrane, trafficking and fusion with a specific acceptor membrane. Prior to the fusion stage, a set of tethering factors allows the vesicle to be in close proximity to the target membrane, thus helping vesicle trafficking organization. Recently, the Transport Protein Particle (TRAPP) complex, a multisubunit tethering complex, was experimentally characterized in Arabidopsis [85], including all 13 mammalian TRAPP subunits as well as an additional plant-specific component, the TRAPP-Interacting Plant Protein (TRIPP). TRIPP plays important roles in trafficking-dependent processes, including the formation of new cell walls and reproductive development [85]. Among the 14 *A. thaliana* TRAPP subunits, we detected 7 homologues (Supplementary Table 1). Besides TRS20, TRIPP and TRS120, which have been shown to interact, were found to be more abundant in mature fruit (Table 1). Both TRIPP and TRS120 are part of TRAPPII, one of a variety of TRAPP modular forms. TRAPPII mutants displayed altered levels of methyl-esterified pectins at cell plates, pointing to a role of this complex in the transport of pectins [86]. Changes in pectin metabolism are one of the hallmarks of the softening and textural changes in melting flesh peach triggered by fruit ripening [87]. Thus, our results help to link cell wall metabolism with vesicle mediated transport of proteins and polysaccharides through the downregulation of TRIPP and TRS120 between mature and ripe fruit.

TRAPPII have also been pointed as a guanine exchange factors (GEFs) for RabA GTPases [88, 89]. RabA GTPases can regulate membrane fusion events and other cell organelle processes once they are recruited to their specific membrane compartment and activated by their cognate RabGEF. Among the proteins downregulated during the mature to ripe fruit transition we found a homologous of RABA5b, a RabA GTPase. Interestingly, the *P. persica* TRIPP, TRS120 and RABA5b displayed a dramatic and linear increase in transcript abundance from 81 to 125 DAP, pointing to a specific role in fruit development for these 3 proteins, and TRIPP-TRS120 TRAPPII as potential GEFs for RABA5b.

Another key component of the vesicle trafficking machinery are the adaptor protein complexes, which participate in the selection of the vesicles specific transported cargo. Using a combination of organelle density gradients with proteome analysis, Pertl-Obermeyer et al. [90] indicated that the adaptor protein complex 4 (AP4) participates in delivering cell wall proteins. AP4 would also be involved in sorting transmembrane proteins to the vacuole [91], being a key component of the post Golgi trafficking. Alterations in the cell wall proteome content as well as active vacuolar sorting have been associated with fruit ripening [14, 92]. We detected two AP4 subunits down-regulated during the transition from mature to ripe fruit (Table 1). Together, our results indicates that an active vesicle mediated-traffic is operating at mature peach fruit, and that during the transition to ripe fruit this process shifts into a less active state, possibly associated with the fruit senescence.

### Transcription factors assessment

Analysis of possible transcription factors (TFs) that could regulate protein-coding genes expressed mostly or specifically during fruit ripening is of high interest given its application in driving the expression of genes of interest in this organ. Their identification could help to uncover the transcription regulation that underlies the extensive changes triggered by fruit ripening. TFs associated to ripening in climacteric fruit have been identified in apricot [93, 94], melon [95], banana [96, 97], tomato [98, 99], papaya [100–102], among others. By 2015, around 1533 TFs were identified in *P. persica* [20], however just a few have been characterized and even less have been associated to the fruit ripening process. MADS-box PrupeSEP1 (Prupe.3G249400) [103], TCP PpTCP.A2 (Prupe.1G272500) [49], homeobox PpHB.G7 (Prupe.1G416800) [104], AP2/ERF PpERF2, PpERF3 and PpERF.E2 (Prupe.5G090800, Prupe.7G194400 and Prupe.3G032300) [54, 105], ARFs PpARF5 (Prupe.1G368300) [106] and EIN3-like PpEIL1 to 5 (Prupe.6G018200, Prupe.2G058400, Prupe.2G058500, Prupe.6G181600, Prupe.2G070300) [105] are among those TFs already characterized and associated with peach fruit ripening. NAC domain TFs were enriched among the TFs that targeted the genes whose respective proteins were more abundant in mature compared to ripe fruit. Several NAC TFs have been reported to be involved in regulating tomato [107], melon [95] and papaya [100] fruit ripening, indicating that NAC TFs role in fruit ripening is evolutionarily conserved [108]. In peach, both ACS and ACO genes, involved in ethylene biosynthesis, display NAC transcription factor binding motifs, and several NAC TFs and key fruit ripening genes display a ripening-specific expression pattern [32]. Protein-coding genes whose products were more abundant in ripe compared to mature fruit also displayed an enrichment as NAC TFs targets, however they were mainly enriched as MYB TFs targets. MYB ripening-related TFs have been characterized in *Lycium ruthenicum* [109], in tomato [110], in papaya [101] and in plum (*P. salicina*) [111], indicating that MYBs might also be key in modulating the peach fruits pigmentation, flavour, and texture changes triggered by the fruit ripening [112].

Just one TF would have as targets the protein-coding genes for both mature and ripe fruit when compared to all 1663 proteins identified in the present work. This protein was a HB40-homolog protein (Prupe.7G149700), which displayed a strong expression peak at the stage of fruit ripening (Fig. 6b). In tomato, the MADS box TF RIPENING INHIBITOR (RIN) has been placed as an early ripening regulator. The best tomato homologous of Prupe.7G149700, the gene Solyc02g085630 (64% identity), was among the RIN targets identified by chromatin immunoprecipitation coupled with DNA microarray analysis (ChIP-chip) [113], indicating that these HB40-homolog proteins could be effectors of RIN.

### Future perspectives

Plant Proteomics analysis has benefited, in the last years, from the increasing development of mass spectrometry (MS), the publication of hundreds of plant genomes (430 to date) [114] and generation of customized bioinformatics tools and pipelines. 1D-gel electrophoresis followed by LC/MS-MS analysis (Fig. 1), has the great advantage of being straightforward to execute and analyze, as well as being among the most robust pipelines for proteomic analysis [115]. Besides gel based, gel free [116], antibody chips [13] and MS-imaging [117] are also available for analyzing plant sample proteome contents. However, the most promising approaches are related to how validated and integrate proteomics data with other omics approaches in order to get a more systemic vision of the processes under evaluation [118]. In this work we integrated proteomics data generated by us and others, as well as different sets of transcriptomic data publicly available to unveil information regarding critical molecular processes involved in the peach fruit ripening.

*P. persica* belongs to the Rosaceae family, whose species have developed a wide array of fruit types, including drupe, pome, drupetum, achene, and achenetum, making it one of the most informative plant family to perform comparative developmental and evolutionary studies [119]. *P. persica* displays a drupe-type of fruit, where a fleshy fruit encloses a lignified endocarp surrounding a seed. The presence of this lignified endocarp imposes a challenge to the proper fruit development and ripening, since the phenylpropanoids allocation must be carefully controlled for the endocarp lignification during development or to the biosynthesis of flavour/aroma compounds in the ripe fruit mesocarp [120]. This characteristic is particular to this kind of fruit, making the extrapolation of what is known in the so far best fleshy fruit model tomato ripening, limited. In addition, as mentioned previously, the tomato ripening regulatory circuit is mainly based on the action of MADS TFs, whereas in peach NAC-type TFs would have a predominant role in controlling the ripening regulatory circuit [32]. Assessing the molecular mechanisms, using integrated omics approaches, of plants with combinations of the slow ripening (SR) allele, which can render plants with unique phenotypes [121], as well as the results of different pre-harvest treatments [67, 122] that affects the fruit quality will help to uncover new key players that are probably particular to drupe-type fruits.

## Conclusions

We have identified 1663 proteins with high confidence using 1D SDS-PAGE fractionation associated to MS-MS detection. Above a quarter of the genes that code for these proteins were preferentially expressed at mature-ripe fruit in terms of developmental stage and tissue, indicating that these proteins were mainly involved in the fruit ripening. The differentially accumulated proteins in mature and ripe fruit identified in this study showed a high correlation with previous transcriptome studies. Differentially accumulated proteins were mainly related to the metabolism of hormones such as ethylene and brassinosteroids, sugar metabolism, cell wall rearrangement, fruit aroma and lipid metabolism.

Mature fruit displayed more changes than ripe fruit, with several changes associated to well characterized pathways, in contrast to ripe fruit, where many changes could not be mapped to coordinated biochemical processes. This indicates that the metabolism of mature fruit is more regulated than the one from ripe fruit, which agrees with the idea that ripening fruit undergoes a coordinated senescence process.

The identification of proteins with a marked differential accumulation during the mesocarp fruit ripening process will help to set experimental designs that could allow a more detailed analysis of the ripening process itself. In the near future, the use of precise and reliable near infrared spectroscopy based non-destructive tools [123] together with the molecular profiling of these proteins could make possible to perform a detailed analysis of the ripening stages SI to SIII, since by now no phenotypical, anatomical, biochemical or morphological parameter can help to discriminate these stages among fruits that display, on the tree, a great variation in terms of maturity stage, hampering this assessment.

## Methods

### Plant material

Regular size fruit were harvested from 8-year-old ‘O’Henry’ trees grown on Nemaguard rootstocks in a commercial orchard located in the Aconcagua Valley, Chile (34^o^17’ W, 70^o^54’ S) [28]. As harvesting index we used the change in fruit ground color, measured using fruit company’s color table. Two postharvest stages were selected for proteome analysis: mature fruit (O1), that consisted on firm fruit taken immediately after harvest (firmness of around 60 N) and in the stage required for packing; and ripe fruit (O2), that consisted on fruit ripen at 20 °C (firmness of around 11 N) [28]. Physiological parameters such as firmness, color, total soluble solids, titratable acidity, respiration rate and ethylene production were measured by Campos-Vargas et al. [28]. Three fruits from each postharvest stage were used for protein extraction and considered biological replicates (Fig. 1a).

### Protein extraction and SDS-PAGE

One milligram of mesocarp tissue from each biological sample was pulverized in liquid nitrogen and transferred to a room temperature tube with 5 mL of protein extraction buffer [124]. This solution was mixed with 5.5 mL of Tris-saturated phenol pH 8.0 and shaken vigorously for 5 min at room temperature, followed by centrifugation at 8500×g for 14 min at 4 °C to achieve phase separation. The phenolic phase was recovered, re-extracted with an equal volume of protein extraction buffer and precipitated for 2 h at − 20 °C by the addition of five volumes of 0.1 M ammonium acetate in methanol at − 20 °C [26]. The precipitated material was collected by centrifugation at 8500×g for 12 min and protein pellets were washed three times with cold ammonium acetate in methanol and once with 80% acetone at − 20 °C. The pellet was dried at room temperature and then solubilized in 300 μL of resuspension buffer which contained 5 M urea, 2 M thiourea, 2% CHAPS (w/v), 2% SB3–10 (w/v), 0.5% ampholites pH 5–7 (v/v) and 0.25% ampholites pH 3–10 (v/v) [125]. Protein yield was determined by Bradford protein assay [126]. All samples were stored at − 80 °C prior to electrophoresis.

SDS-PAGE was performed on 12% polyacrylamide gels casted on a BIO-RAD Mini-PROTEAN Tetra cell with 1 mm spacer plates. A hundred and fifty micrograms of protein samples were mixed with 10 μL Coomassie blue 5X loading buffer and run at 80 V until the dye front reached the bottom of the gel. Band visualization was achieved by staining the gels with colloidal Coomassie G250. Only one sample was run per gel. Each lane loaded with proteins was excised and divided into ten slices of 4 mm × 4 mm × 1 mm (Fig. 1b).

### Experimental LC/MS/MS

Gel slices were digested in-gel according to Shevchenko et al. [127] with modifications. Gel bands were dehydrated using 100% acetonitrile and incubated with 10 mM dithiothreitol in 100 mM ammonium bicarbonate (pH 8) at 56 °C for 45 min, dehydrated again and incubated in the dark with 50 mM iodoacetamide in 100 mM ammonium bicarbonate for 20 min. Gel bands were then washed with ammonium bicarbonate and dehydrated again. Sequencing grade modified trypsin was prepared to a final concentration of 0.01 μg μL^− 1^ in 50 mM ammonium bicarbonate and 50 μL of this solution was added to each gel band. Bands were then incubated at 37 °C overnight. Peptides were extracted from the gel by water bath sonication in a solution of 60%ACN/1%TCA and vacuum dried to approximately 2 μL. Peptides were then re-suspended in 2% acetonitrile/0.1% trifluoroacetic acid to 20 μL. From this, 10 mL were automatically injected by a Waters nanoAcquity Sample Manager and loaded for 5 min onto a Waters Symmetry C18 peptide trap (5 μm, 180 μm × 20 mm) at 4 μL/min in 5%ACN/0.1% formic acid. The bound peptides were then loaded onto a Waters BEH C18 nanoAcquity column (1.7 μm, 100 μm × 100 mm) and eluted for 35 min with a gradient of 5%B to 30%B in 24 min, ramped up to 90%B at 25 min and held for 1 min, then dropped back to 5%B at 26.1 min using a Waters nanoAcquity UPLC (Buffer A = 99.9% water/0.1% formic acid; Buffer B = 99.9% acetonitrile/0.1% formic acid) with a constant flow rate of 0.8 μL min^− 1^.

Eluted peptides were sprayed into a ThermoFisher LTQ Linear Ion trap mass spectrometer outfitted with a MICHROM Bioresources ADVANCE nano-spray source. The top five ions in each survey scan were then subjected to data-dependent zoom scans followed by low energy collision induced dissociation (CID) and the resulting MS/MS spectra were converted to peak lists in BioWorks Browser v3.2 (www.thermo.com) using the default LTQ instrument parameters. Peak lists were searched against a custom database containing protein sequences from *Prunus persica* whole genome assembly, v1.0 and common laboratory contaminants (downloaded from NCBI, www.ncbi.nlm.nih.gov) using the Mascot searching algorithm, v2.3 (www.matrixscience.com). Mascot parameters for all databases were as follows: allow up to two missed tryptic sites; fixed modification of carbamidomethyl cysteine; variable modification of oxidation of methionine; peptide tolerance of +/− 200 ppm; MS/MS tolerance of 0.6 Da; peptide charge state limited to + 2/+ 3 (Fig. 1c). Translation from v1.0 genome annotation to version 2.1 was achieved by using data provided by Phytozome v12 [128] and by manual analysis of the peptides characterized.

### Data processing

The Mascot output was analyzed using Scaffold v4.8.2 (www.proteomesoftware.com) to statistically support protein identifications. Assignments were validated in Scaffold according to the following criteria: Protein Threshold - 1.0% FDR; Minimum number of peptides - 1; Peptide threshold - 0.1% FDR. To be able to accurately determine changes in protein levels, average total ion chromatograms (Average TIC) was retrieved from Scaffold for each protein assessed in each of the six samples under evaluation [40]. Average TIC for each protein was estimated using only those peptides identified with high confidence (Fig. 1d). Supporting material and protein datasets has been deposited to MassIVE platform under accession number MSV000086519 or ftp://massive.ucsd.edu/MSV000086519/.

### Data pre-treatment

To circumvent the issue of missing data, average values were calculated for those proteins that had only two biological replicates per biological condition (mature or ripe) and were used as third replicate. All the following analyses were thus performed with proteins which had at least two data points in O1 and O2, being the other proteins discarded. Protein data was scaled by using pareto scaling [129], normalized using Quantile Normalized procedure and centered at zero by subtracting the mean value, using InfernoRDN (version 1.1.5970.31895) [130]. This allowed the data to be transformed to achieve a close to normal distribution, required for many statistical analyses of variance (Supplementary Fig. 1). Q-Q and Box-plot graphs were also performed using InfernoRDN.

### Assessment of the physicochemical characteristics of proteins

Proteins derived from the primary transcript of each of the 26,973 protein-coding genes (Ppersica_298_v2.1.protein_primaryTranscriptOnly.fa) were retrieved from Phytozome v12 [128] and were assessed using the Peptides R Package [131]. The following parameters were evaluated for each protein: length, molecular weight (MW, in Daltons), charge (pH = 7, pKscale = “Lehninger”), protein stability (instability index), and hydrophobicity (KyteDoolittle and Guy scale). Mean values and Gini’s mean difference, a measure of variability that is robust even for non-normal data distributions, were computed using the Hmisc R Package.

### Evaluation of protein differential accumulation

To detect differences in protein accumulation in mature and ripe fruits, a two-sample t-statistics with equal variance was performed for each protein/gene under analysis using the Bioconductor ‘multtest’ package followed by the “ABH” (Adaptive Benjamini-Hochberg) correction procedure, with a cutoff value of 0.1.

### Gene ontology and pathways analysis

Gene ontology analysis was performed using Phytomine (https://phytozome.jgi.doe.gov/phytomine/begin.do). The Benjamini-Hochberg test, with a “max *p*-value” set to 0.05, was used for Multiple Testing Corrections. Redundant GO terms were removed with the webtool REVIGO [132], using the following parameters: 1. Allowed similarity: small (0.5); 2. GO categories associated to: *p*-values; 3. GO term sizes database: *Arabidopsis thaliana*; 4. Semantic similarity measure to use: SimRel. Next, CirGO [133] was used to plot a concise version of the GO analysis.

Pathway diagrams were built by first running the program DeepEC [134] to annotate the enzymes included in the 26,873 protein-coding genes from the *P. persica* genome version 2.1. Next, this annotation was used to generate the input file for the program Pathway Tools [135], which was used to infer the pathways and reactions from *P. persica* (Supplementary table 1).

### Principal component analysis (PCA)

PCA analysis was run on normalized and centered data using the Explore/PCA tool from InfernoRDN [129].

### Transcriptional analysis of the gene expression omnibus dataset GSE71561

Data from 13 peach samples, which included six samples from fruit seeds (days 41, 54, 69, 83, 111 and 125 after full bloom – DAFB), the same six samples from fruit mesocarp and one sample from flowers from the mid-season Fantasia variety, was downloaded from the GSE71561 dataset. Thus, samples covered until the commercial ripening stage.

The microarray data was log2 transformed following the protocol available from the online tool GEO2R [136]. This data, representing 29,045 genes, was used to generate heatmaps using the “superheat” R package [137]. The main set parameters were: clustering.method = “hierarchical”, dist.method = “maximum”, linkage.method = “complete”. In order to avoid dealing with probes that target the same gene, all genes were ranked according to their average abundance, and then those genes with repeated measures and lower average abundances were removed. Subsets of this data, such as expression patterns from selected protein-coding genes were also plotted as heatmaps using the same parameters.

In addition, data from mesocarp tissue was used to contrast the expression of genes in the 125 DAFB stage against the remaining five mesocarp samples (M_125 vs M_41, M_125 vs M_54, M_125 vs M_69, M_125 vs M_83, M_125 vs M_111). This task was performed using the Limma R package [138], following the protocol available from the online tool GEO2R [136] and using the “decideTests” function. To improve the statistical confidence of the analysis, the probes that targeted the same gene had to display the same “decideTests” results in order to be considered for further analysis. This approach reduced the original dataset size from 29,045 genes to 18,074 genes (62.2%). These 18,074 genes included information from 1136 protein-coding genes (68.3%) present in our proteomics dataset.

### Retrieval and analysis of transcription factors regulatory elements

Transcription factors (TFs) that possessed over-represented targets in the protein set differentially accumulated in mature and ripe fruits and among all the proteins assessed in this study, were retrieved from the Plant Transcription Factor Database (PlantTFDB) [139], using the “Regulation Prediction” tool from the Plant Transcriptional Regulatory Map. Next, Phytomine (https://phytozome.jgi.doe.gov/phytomine/begin.do) was used to retrieve protein domains enriched for these TFs. The Benjamini-Hochberg test, with a “max *p*-value” set to 0.05, was used for Multiple Testing Corrections.

### Gene family number analysis

*P. persica* gene family numbers were retrieved from PLAZA 4.0 [140], using the respective *P. persica* genome v2.1 ID as identifier.

## Supplementary Information

**Additional file 1: Supplementary Fig. 1.** Data pre-treatment. In order to have an insight about the data distribution, Q-Q plots were generated using as input the raw (A), versus imputed-scaled-centered protein abundance data (C). As a result from pre-treatment data, the Q-Q plot curve fitted much closer to the normal expectation curve (straight red diagonal line) than with the raw data. Boxplots of raw (B) vs treated data (D) further highlighted how the data became centered and scaled after been pre-treated.

**Additional file 2: Supplementary Fig. 2.** Proteome bias assessment. Protein parameters were compared among *P. persica* primary transcripts’ proteome (left panels), current proteome (middle panels) and a mesocarp-derived proteome extracted from juicy and mealy fruits from the Spring Lady variety (right panels) [30]. Panels I to III contrast proteomes in terms of length and molecular weights (MW). Panels IV to VI contrast proteomes in terms of charge and protein stability based on its amino acids (instalindex). Panels VII to IX contrast proteomes in terms of hydrophobicity, using two scales: KyteDoolittle and Guy. Each dot in each graph represents the intersection of the values one protein has for the two parameters under evaluation. The distributions of values for each of these two parameters are shown above and at the right side of each panel as density plots.

**Additional file 3: Supplementary Fig. 3.** Hierarchical clustering of all genes transcriptionally characterized in the GSE71561 dataset and a subset related to sorbitol biosynthesis. Transcriptional information (average of three replicates of log2 normalized fluorescence intensity values) from 29,045 genes from the peach fruit genome 1.0, assessed in 13 conditions, was displayed using hierarchical clustering and the following conditions: clustering.method = “hierarchical”, dist.method = “maximum”, linkage.method = “complete“(panel I). Using the same stages, data from genes encoding a putative sorbitol transporter family is also depicted (panel II). S_45 to S_125, seed samples at 45, 54, 69, 83, 111 and 125 days after full bloom (DAFB); M_45 to M_125, mesocarp samples at 45, 54, 69, 83, 111 and 125 DAFB; F - flower samples.

**Additional file 4: Supplementary Fig. 4.** Comparison of the O’Henry fruit mesocarp proteome characterized by 2D gel vs 1D gel analysis. (a) Mesocarp proteins from mature and ripe O’Henry fruits assessed by 2D-gels had 164 spots that could be quantified [27]. Among these 164 spots, 43 were identified by mass spectrometry analysis and, therefore, were contrasted with the current proteome under analysis. (b) Among these 43 proteins, 16 had accumulation profiles similar to the ones assessed in the current work (“match”), whereas 27 had different patterns (“did not match”).

**Additional file 5.**

**Additional file 6.**

**Additional file 7.**

## Data Availability

Supporting material and protein datasets has been deposited to Mass Spectrometry Interactive Virtual Environment (MassIVE) under accession number MSV000086519 or ftp://massive.ucsd.edu/MSV000086519/. Data generated during analysis are included in the manuscript as supplementary files.

## Abbreviations

ACHT4: Atypical cysteine/histidine-rich thioredoxin 4ACO - ACC oxidase
ACS: ACC synthase
AP4: Adaptor protein complex 4
APS1: Glucose-1-phosphate adenylyltransferase small subunit
BSK: BRASSINOSTEROID-SIGNALING KINASE
CXE: Carboxylesterase
DAFB: Days after full bloom
F6P: Fructose 6-phosphate
FAD1: Fatty acid desaturase 1
FERLs: Feronia-like receptor kinases
GO: Gene Ontology
MS: Mass spectrometry
PA: Phosphatidic acid
PCA: Principal Component Analysis
PEP: Phosphoenolpyruvate
PG: Polygalacturonase
PLD: Phospholipase D
RAFS: Raffinose synthase
SAM: S-Adenosyl methionine
SBE: 1,4-alpha-glucan-branching enzyme
SOT: Sorbitol transporter
TFs: Transcription factors
TIC: Total ion chromatograms
