# Supplementary figures and images for: Shotgun proteomics of peach fruit reveals major metabolic pathways associated to ripening
Source: BMC Genomics. 2021 Jan 6;22:17. doi: 10.1186/s12864-020-07299-y (PMC7788829; doi:10.1186/s12864-020-07299-y)

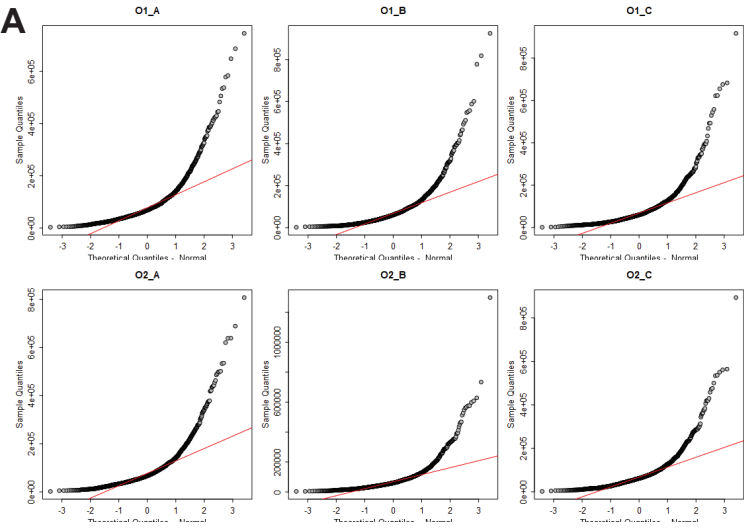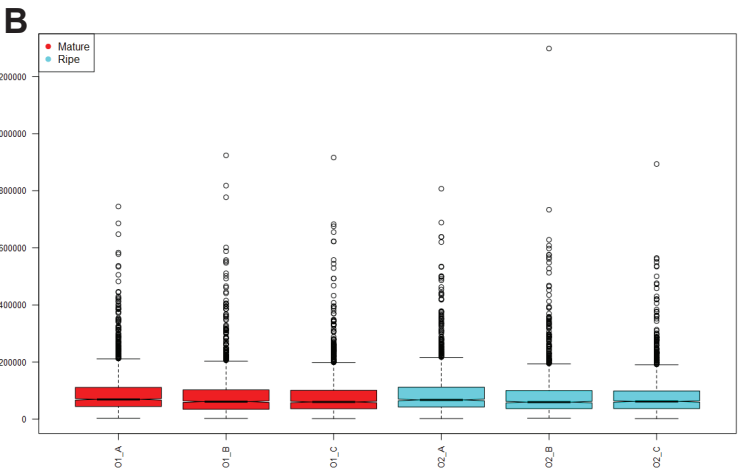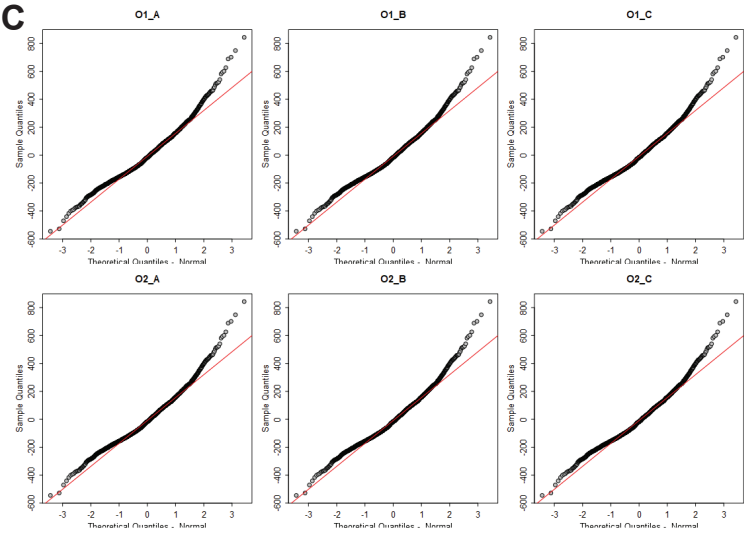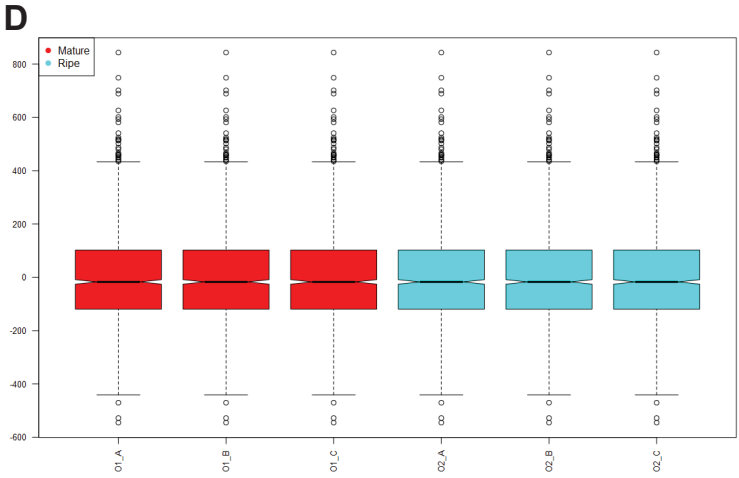

Supplement: Supplementary file 1 — Additional file 1: Supplementary Fig. 1. Data pre-treatment. In order to have an insight about the data distribution, Q-Q plots were generated using as input the raw (A), versus imputed-scaled-centered protein abundance data (C). As a result from pre-treatment data, the Q-Q plot curve fitted much closer to the normal expectation curve (straight red diagonal line) than with the raw data. Boxplots of raw (B) vs treated data (D) further highlighted how the data became centered and scaled after been pre-treated. [file 12864_2020_7299_MOESM1_ESM.pdf]

I

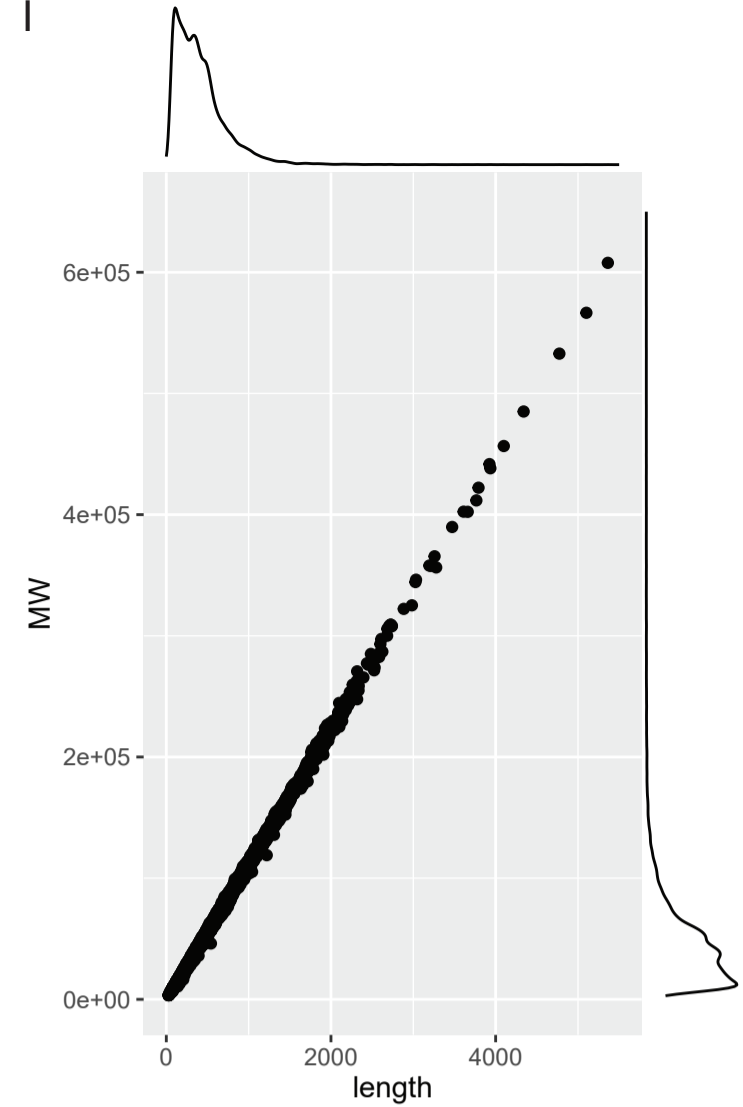

II

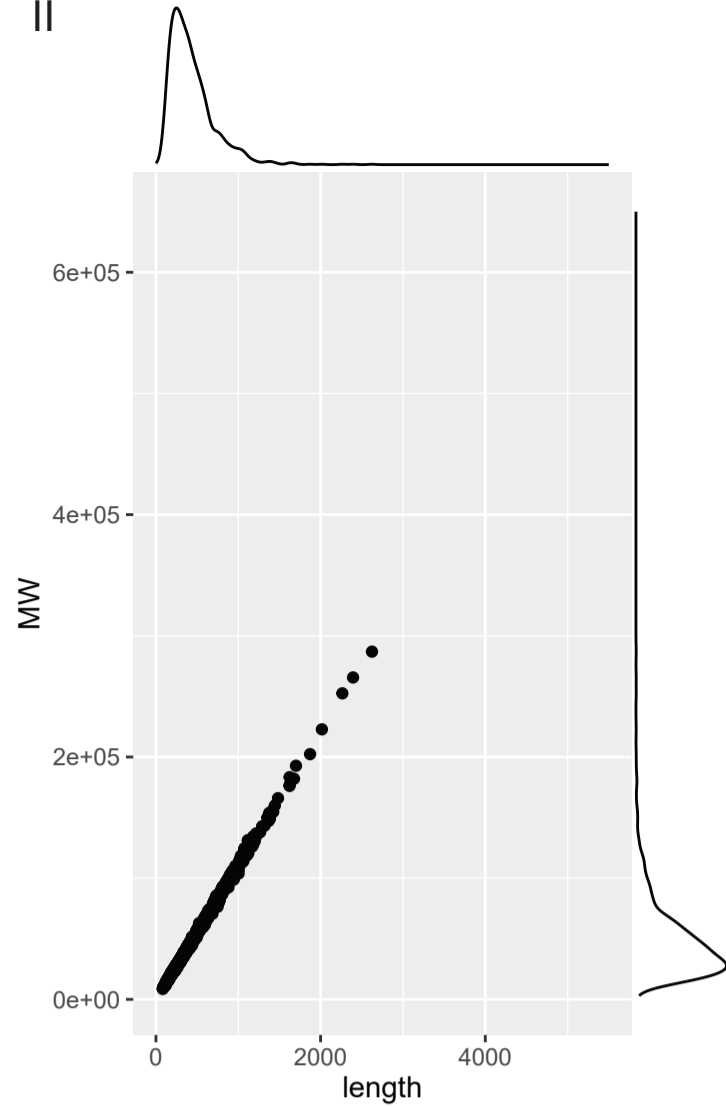

III

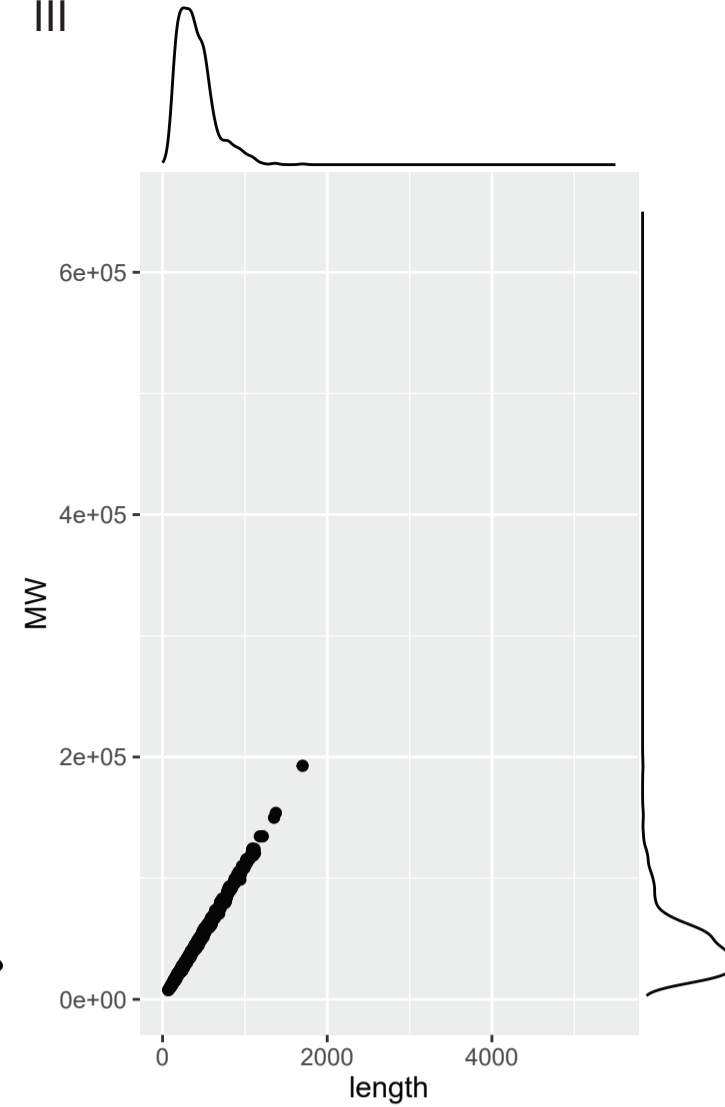

IV

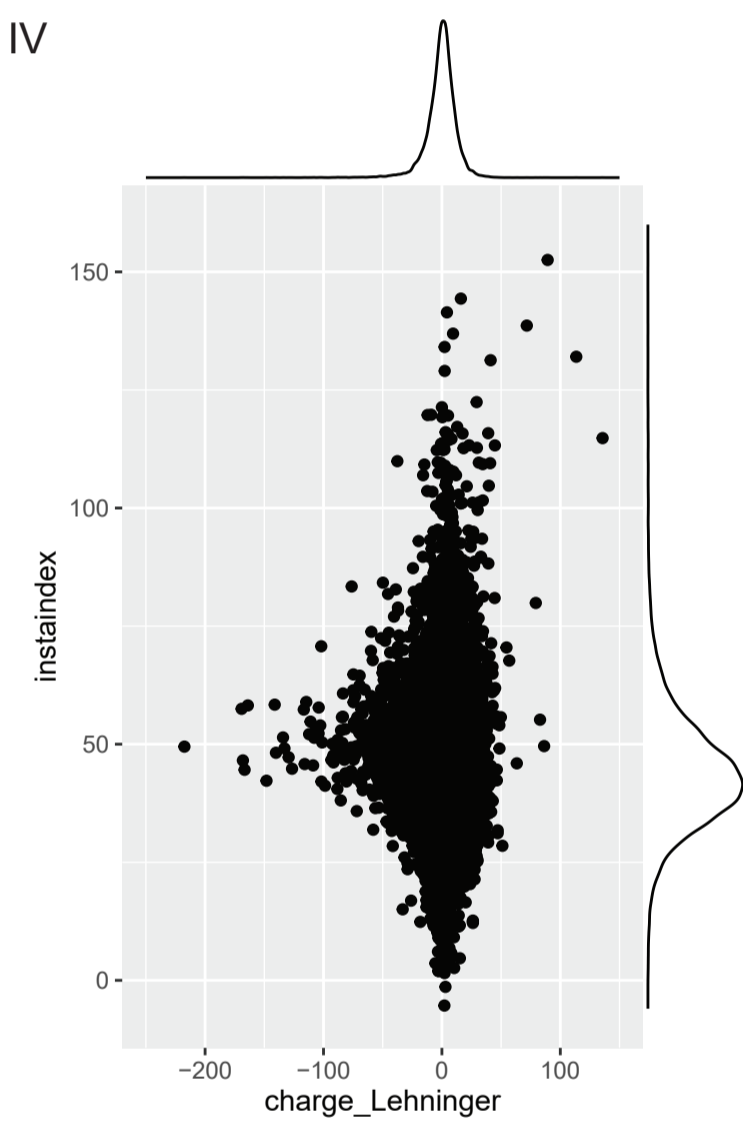

V

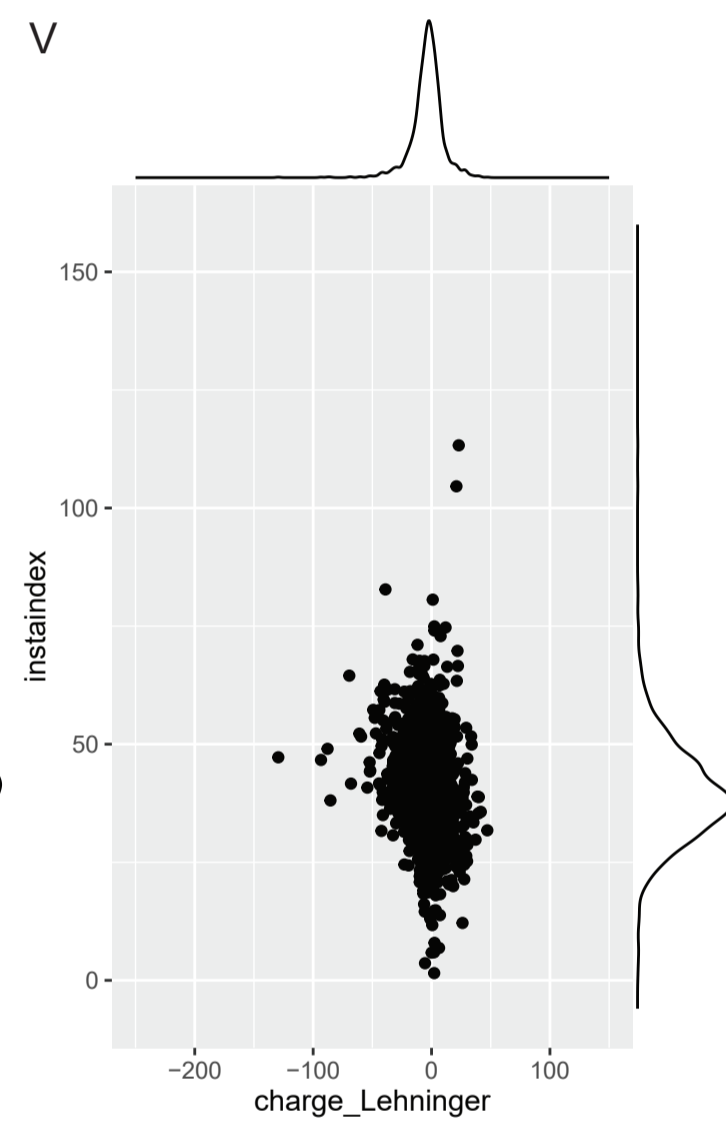

VI

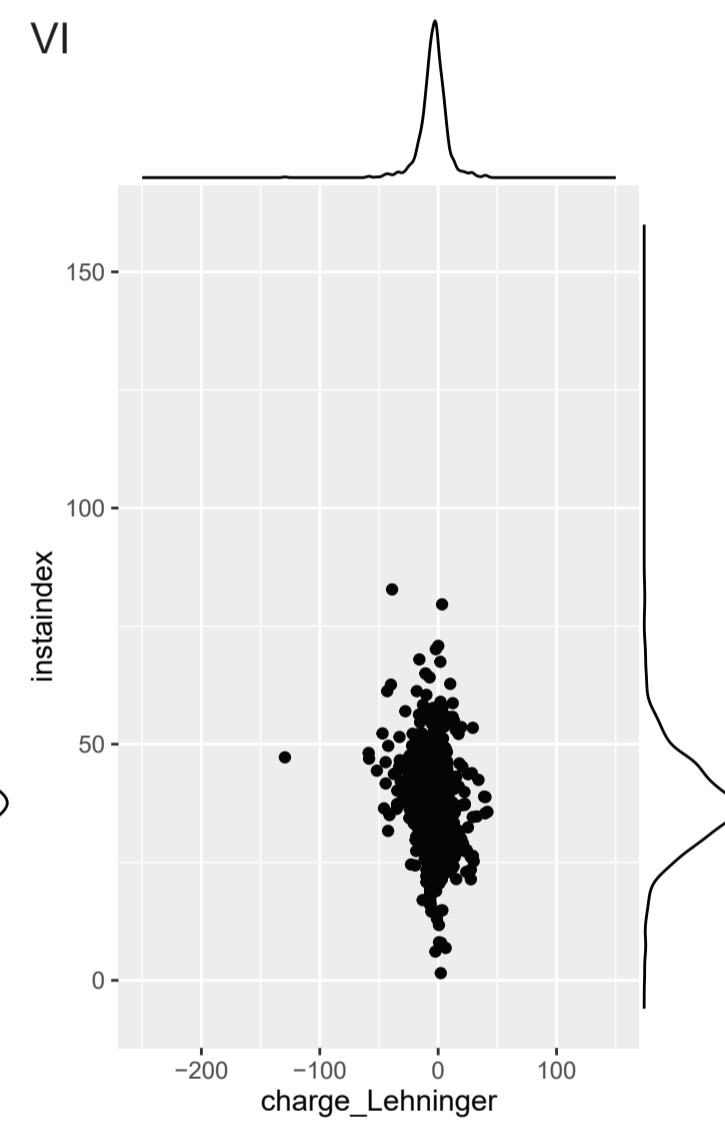

VII

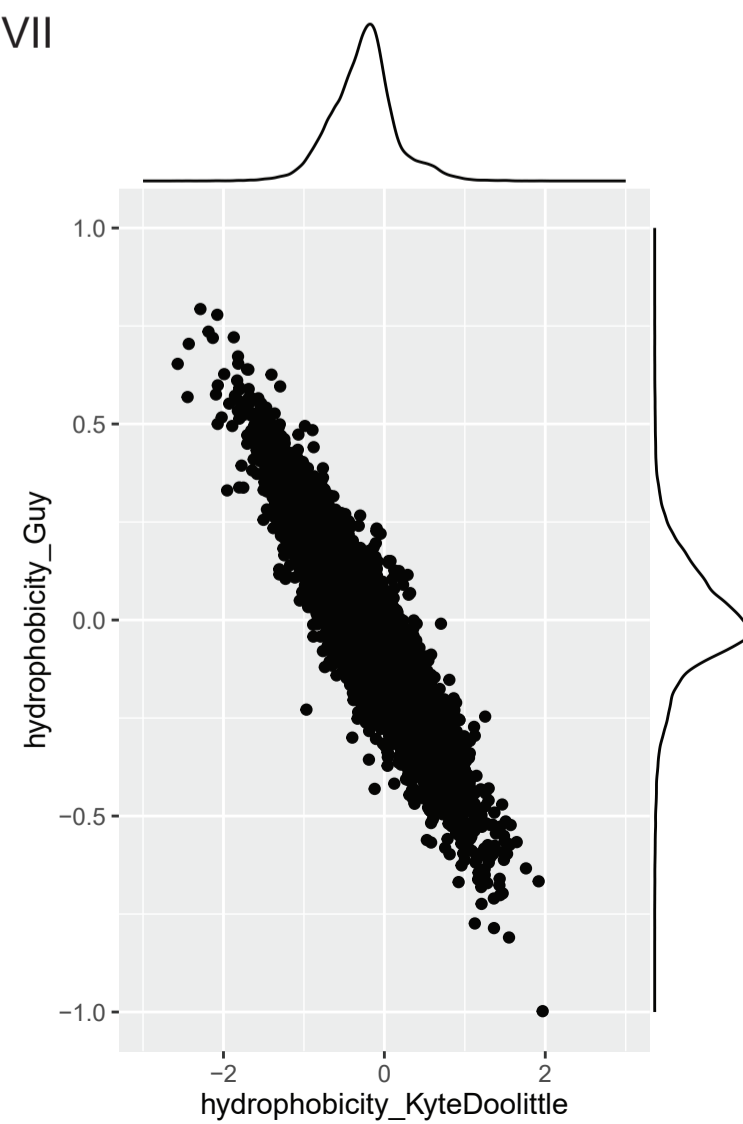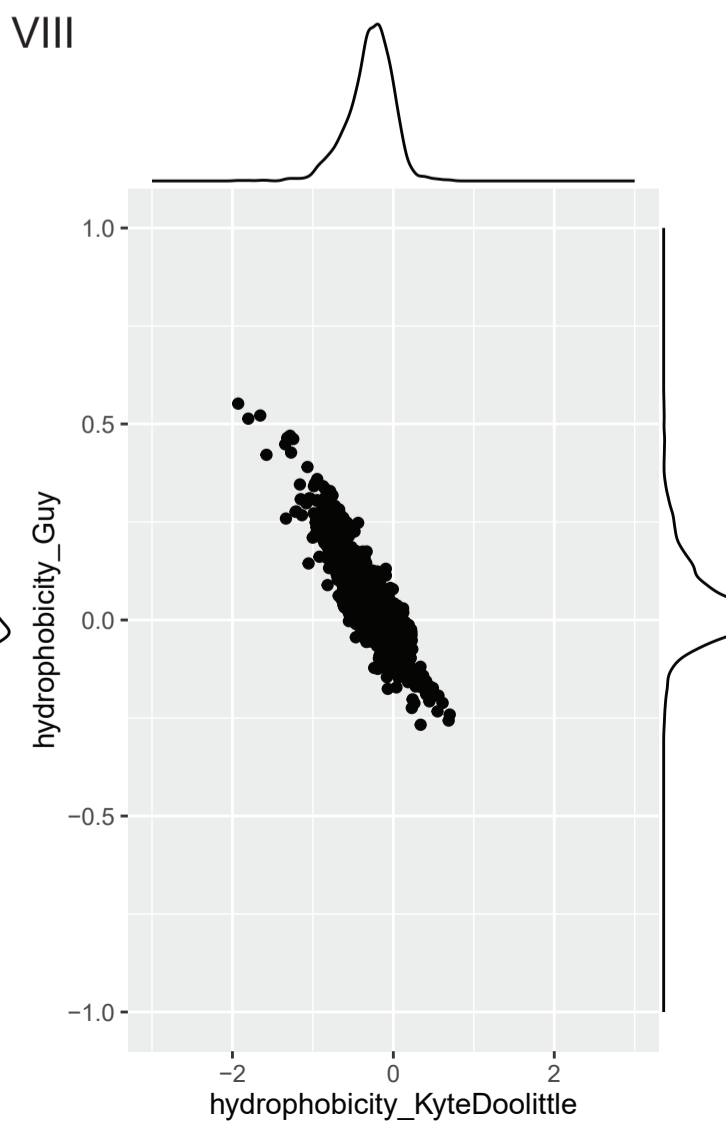

IX

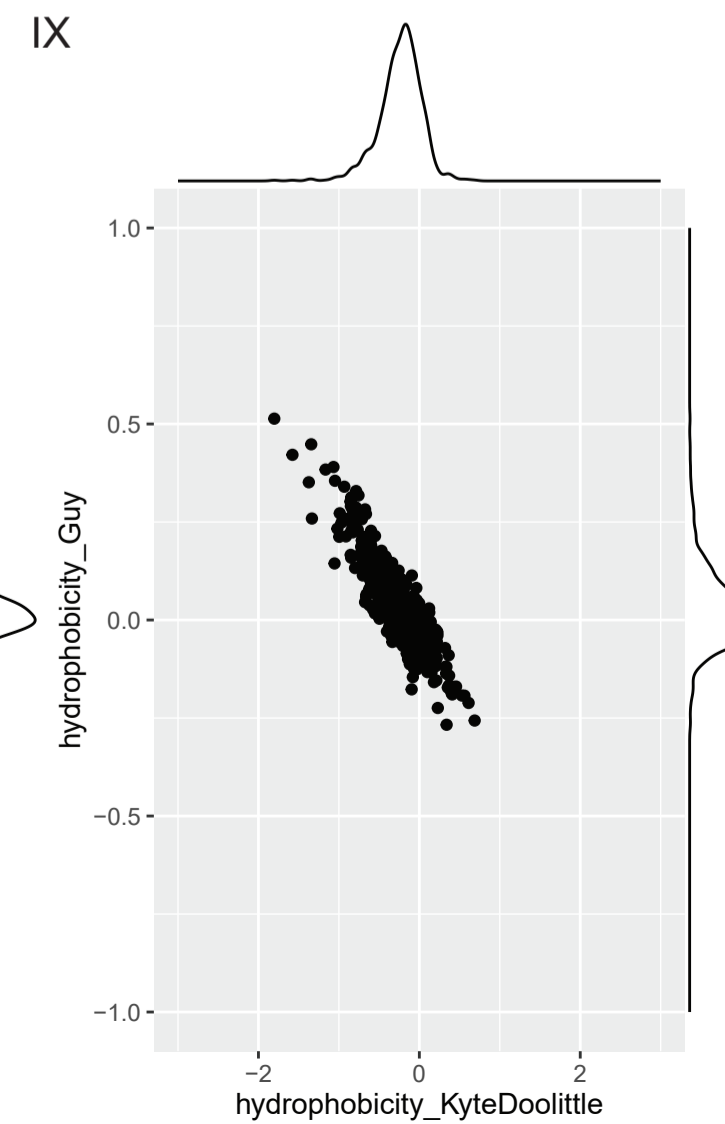

Supplement: Supplementary file 2 — Additional file 2: Supplementary Fig. 2. Proteome bias assessment. Protein parameters were compared among P. persica primary transcripts’ proteome (left panels), current proteome (middle panels) and a mesocarp-derived proteome extracted from juicy and mealy fruits from the Spring Lady variety (right panels) [30]. Panels I to III contrast proteomes in terms of length and molecular weights (MW). Panels IV to VI contrast proteomes in terms of charge and protein stability based on its amino acids (instalindex). Panels VII to IX contrast proteomes in terms of hydrophobicity, using two scales: KyteDoolittle and Guy. Each dot in each graph represents the intersection of the values one protein has for the two parameters under evaluation. The distributions of values for each of these two parameters are shown above and at the right side of each panel as density plots. [file 12864_2020_7299_MOESM2_ESM.pdf]

A

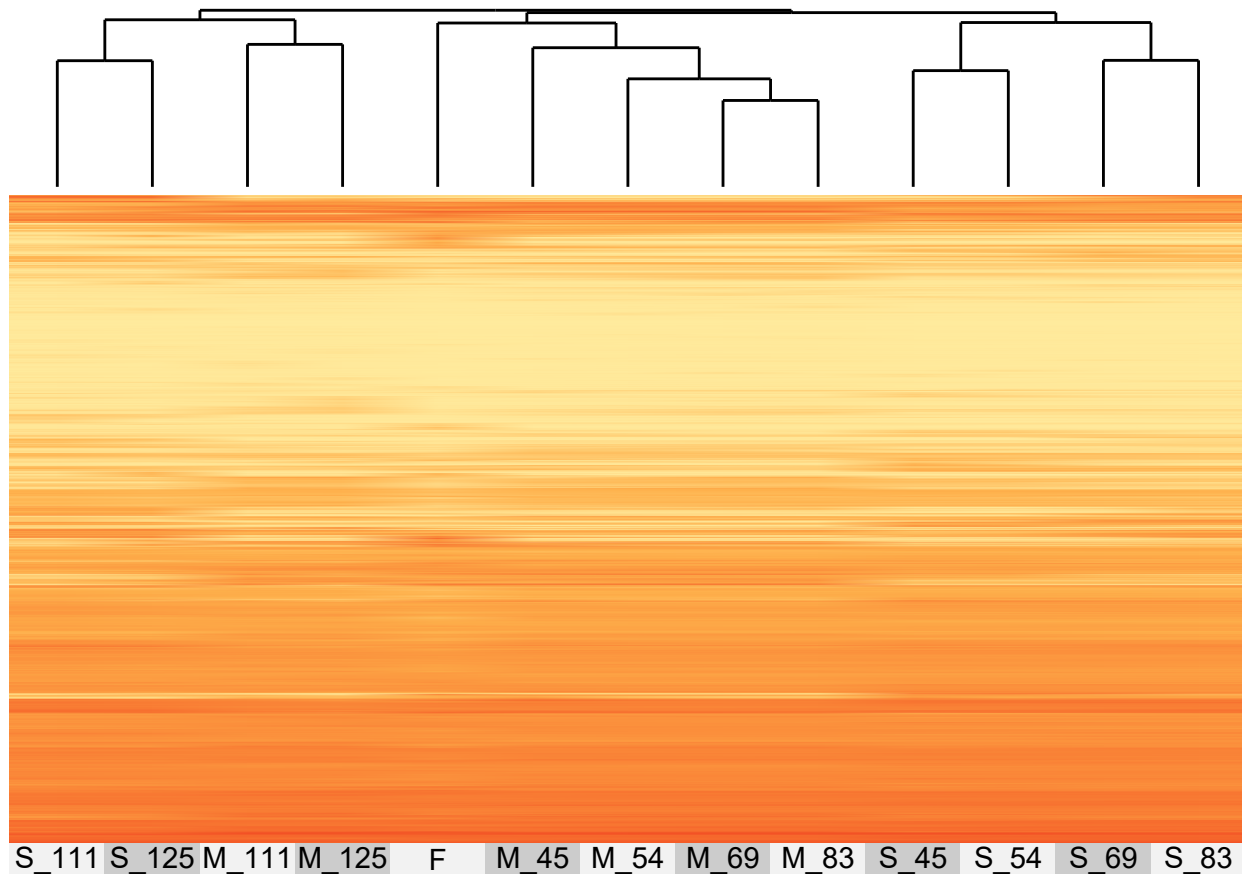

B

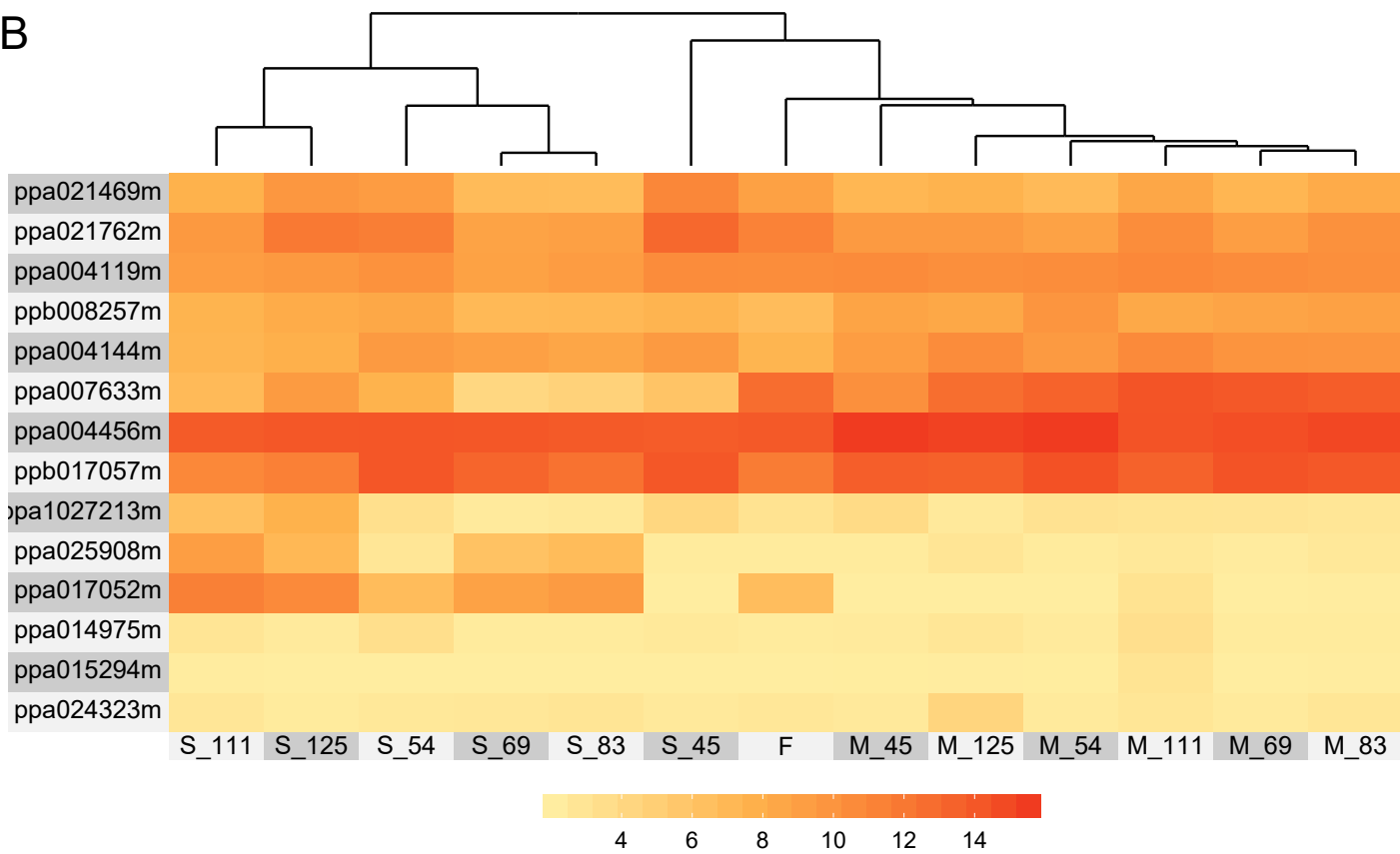

Supplement: Supplementary file 3 — Additional file 3: Supplementary Fig. 3. Hierarchical clustering of all genes transcriptionally characterized in the GSE71561 dataset and a subset related to sorbitol biosynthesis. Transcriptional information (average of three replicates of log2 normalized fluorescence intensity values) from 29,045 genes from the peach fruit genome 1.0, assessed in 13 conditions, was displayed using hierarchical clustering and the following conditions: clustering.method = “hierarchical”, dist.method = “maximum”, linkage.method = “complete“(panel I). Using the same stages, data from genes encoding a putative sorbitol transporter family is also depicted (panel II). S_45 to S_125, seed samples at 45, 54, 69, 83, 111 and 125 days after full bloom (DAFB); M_45 to M_125, mesocarp samples at 45, 54, 69, 83, 111 and 125 DAFB; F - flower samples. [file 12864_2020_7299_MOESM3_ESM.pdf]

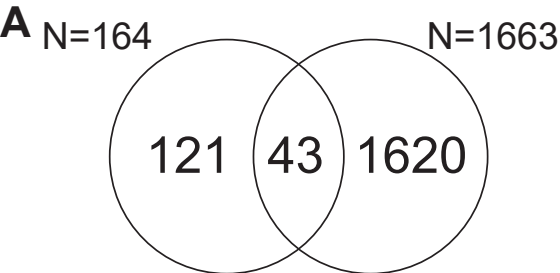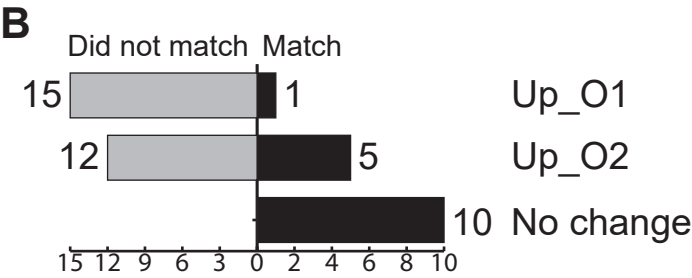

Supplement: Supplementary file 4 — Additional file 4: Supplementary Fig. 4. Comparison of the O’Henry fruit mesocarp proteome characterized by 2D gel vs 1D gel analysis. (a) Mesocarp proteins from mature and ripe O’Henry fruits assessed by 2D-gels had 164 spots that could be quantified [27]. Among these 164 spots, 43 were identified by mass spectrometry analysis and, therefore, were contrasted with the current proteome under analysis. (b) Among these 43 proteins, 16 had accumulation profiles similar to the ones assessed in the current work (“match”), whereas 27 had different patterns (“did not match”). [file 12864_2020_7299_MOESM4_ESM.pdf]
